# Who Cries Wolf, and When? Manipulation of Perceived Threats to Preserve Rank in Cooperative Groups

**DOI:** 10.1371/journal.pone.0073863

**Published:** 2013-09-12

**Authors:** Pat Barclay, Stephen Benard

**Affiliations:** 1 Department of Psychology, University of Guelph, Guelph, Ontario, Canada; 2 Department of Sociology, Indiana University, Bloomington, Indiana, United States of America; Universidad Carlos III de Madrid, Spain

## Abstract

People perform greater within-group cooperation when their groups face external threats, such as hostile outgroups or natural disasters. Researchers and social commentators suggest that high-ranking group members manipulate this “threat-dependent” cooperation by exaggerating threats in order to promote cooperation and suppress competition for their position. However, little systematic research tests this claim or possible situational moderators. In three studies, we use a cooperative group game to show that participants pay to increase others’ perceptions of group threats, and spend more on manipulation when holding privileged positions. This manipulation cost-effectively elicits cooperation and sustains privilege, and is fostered by *competition* over position, not only position per se. Less cooperative people do more manipulation than more cooperative people do. Furthermore, these effects generalize to broader definitions of privilege. Conceptually, these results offer new insights into an understudied dimension of group behavior. Methodologically, the research extends cooperative group games to allow for analyzing more complex group dynamics.

## Introduction

In George Orwell’s novel *1984*, the ruling party perpetuates apparent war to create solidarity and support for their leader and to suppress questioning of their regime. Although this example is fictional, many real group leaders – political and otherwise – have been accused of creating simulated or actual threats to the group to rally support for their leadership. In the United States, for example, such allegations have been leveled at presidents from both major parties [1]–[2]. More broadly, some commentators argue that leaders commonly exaggerate threats as a political strategy for generating in-group solidarity [3]–[4]. Given the well-documented tendency of individuals to unite and cooperate in the face of a common threat ([5]–[10], reviewed by [11]–[12]), groups might be especially vulnerable to this type of manipulation. Specifically, by encouraging group members to direct resources towards coping with a threat, high-ranking group members could reduce the capacity of individuals to invest those resources in competing for rank within the group. However, despite widespread allegations of threat manipulation in popular culture, it has received little systematic empirical attention from psychologists.

As a result, we know little about the conditions under which individuals will deceive their groups about the likelihood of potential threats, and the degree to which such threats are actually effective. This is surprising, given that threat manipulation holds theoretical implications for the study of group processes, power, and cooperation, as well as practical implications for the study of politics and other real-world competitions for rank. To address this gap, we test hypotheses regarding the antecedents and effects of threat manipulation in three studies. We show that high-ranking individuals manipulate threats more than low-ranking individuals, even when all individuals can manipulate and “rank” is thinly defined. We further show that threat manipulation is cost effective and is driven by *competition* over rank, not only possession of high rank. Our results demonstrate the existence of threat manipulation, as well as key moderators.

### The Puzzle of Cooperation

To understand threat manipulation, we first elaborate on its theoretical rationale. For high-ranking persons, inducing other group members to cooperate poses a challenge. Indeed, understanding why people cooperate within groups is a central puzzle in many disciplines [13]–[17]. The puzzle of cooperation is that it increases group productivity, success, and even survival, but the associated costs tend to decrease cooperators’ net payoffs relative to non-cooperators [18]–[19]. For example, defending one’s group during intergroup conflict increases the group’s chances of survival, but puts oneself at greater risk than those who do not contribute to defending the group.

Additionally, costly cooperation reduces an individual’s ability to compete over hierarchical position within groups, e.g. wealth, dominance, or other forms of social rank. Many real-world groups – including political parties, firms, labor unions, gangs, and non-human societies – are characterized by hierarchies, in which high-ranking members have greater access to resources, but also face competition for their positions from low-ranking members [12], [18], [20]. Competing for position in a hierarchy can be costly, requiring time, effort, or other resources. As such, there exists a trade-off between investing in cooperation to produce collective benefit, versus investing in within-group competition in a hierarchy [18], [21]–[22]. For example, there are often power struggles within political parties and coalitions, but such internal battles can leave them too weak to contest a general election successfully (for a discussion of these dynamics, see [23]). This trade-off is most pronounced in hierarchies based on dominance (i.e. rank based on raw competitive power) such as those found in dictatorships [24], youth gangs [20], among prisoners [25], and many non-human societies [26], but may even exist within some prestige-based hierarchies based on respect and social skills (for the distinction between dominance and prestige, see [27]; our research focuses more on the former.

### External Threats Promote within-Group Cooperation

Despite the disincentives for costly cooperation, individuals can benefit from it under some circumstances. Notably, costly cooperation can be individually beneficial if groups risk failure due to external threats, such as harsh environmental conditions, natural disasters, or intergroup competition [14], [16], [28]. Group membership provides direct and indirect benefits in many species [17], [29], giving individuals a stake in their group’s continued existence [17], [30]–[31]. When the existence of a valued group is threatened, cooperation can be individually beneficial if it aids group well-being, provided that intra-group competition is not too intense [14], [19], [21]–[22].

As a result, an individual’s cooperation level should vary according to the relative pressures of intra- and intergroup competition. Such “threat-dependent” strategies – cooperating when one’s group faces extinction but free-riding when the group prospers – may play an important role in cooperation [30], [32]–[33]. Individuals benefit from cooperating in response to group threats because they benefit from group survival under unstable conditions, yet benefit from selfishness under stable conditions [30]. Because it is beneficial, this “threat-dependent cooperation” could be rationally chosen, learned via reinforcement, or evolved via natural selection (we are agnostic as to the specific developmental process). Accordingly, humans cooperate more when faced with group failure [16], [34] or intergroup competition [5], [9], [12], [32], [35]–[36]; similar effects have been found in non-humans [33]. However, comparatively little work has examined how others might exploit this behavior.

### Do People Manipulate Apparent Threats?

We suggest that the tendency of groups to cohere when threatened leaves them vulnerable to exploitation by manipulation of apparent threats. Given that cooperation decreases one’s ability to compete for group resources, yet increases the total available group resources, group members benefit from manipulating others’ perceptions of group threats in order to encourage cooperation and reduce others’ competitiveness [8], [30]. Such manipulation of apparent group threats could occur in any social system (human or non-human) in which there is competition over positions in a hierarchy, especially when individuals benefit from cooperating to prevent adverse group outcomes, because this manipulation allows one to claim a larger relative share of a larger public good. The methods of manipulation could include reminders of past attacks or disasters [10], [37] or mortality [38], use of “us versus them” language [39], appearances of constant vigilance [10], (un)conscious overestimates and over-responses to potential threats, or even actually increasing intergroup conflict or other threats [39]. Non-humans could use false appearances of vigilance, or false alarm calls [40].

### Manipulation and Privileged Group Members

In most popular accounts of threat manipulation, leaders manipulate followers. Why should we expect high-ranking group members to manipulate threats to a greater degree than low-ranking group members? Although definitions of rank and privilege vary (see below), in theory those who hold privileged positions particularly benefit from manipulating perceived threats [30] to increase group productivity and suppress competition over relative position. This is because they (a) have greater access to group resources by definition [18], [27], (b) have the most to lose by intra-group competition, and (c) can disrupt alliances and “revolutionary coalitions” [41] against themselves. In contrast, the benefits of manipulation are mixed for low-ranking participants. Those lower in the hierarchy may also benefit somewhat from increased group cooperation, but due to their weaker position they must often collaborate in order to successfully compete with a higher-ranking group member [41]. For those low in the hierarchy, the risk of manipulating threats is that they will undermine coalition formation, by encouraging other low-ranking group members to direct resources towards the apparent threat rather than in efforts to supplant the privileged individuals.

In addition to alleged political examples [1]–[2], there is anecdotal evidence suggesting that other types of leaders or privileged persons will manipulate others’ perceptions of group threats [6], [42]. However, there is little experimental evidence, let alone investigations as to the factors that promote such behavior. More generally however, our argument is consistent with recent work suggesting that leaders will act against their group’s interest. For example, recent work has shown that holding a privileged position within a group increases tendencies associated with selfish behavior, including disinhibition [43], objectification of others [44], and decreased perspective-taking ([45], reviewed by [46]). In addition, Maner and Mead [47] found that some people who were assigned leadership positions were willing to withhold helpful information from the group or exclude potential competitors, even when doing so reduced the group’s productivity, though this tendency disappeared under intergroup competition. Thus, holding a privileged position in a group can increase selfish behavior (though does not uniformly do so, [48]).

Thus, while research is consistent with our argument, it remains unknown whether high-ranking group members will create false threats to suppress within-group competition and promote within-group cooperation, and if so, what situational factors moderate this behavior. Unlike the findings described above, threat manipulation requires that privileged individuals actually deceive their fellow group members, and can result in the collapse of the entire group if it results in a failure to cooperate. In addition, our work examines cases in which it is personally costly to manipulate threats, whether this provision of false information is effective, whether it pays off, and how competition for rank will affect group cooperation and stability.

### The Current Studies

We designed an experimental game to test the predictions that:

- People pay to increase (rather than decrease) others’ perceptions of group threats;
- Those possessing high (dominance-based) rank will do so more than those possessing low rank;
- Such strategies are cost-effective at eliciting cooperation and suppressing competition;
- *Competition* over high-ranking positions increases manipulation, not only possession of these positions themselves (Study 2);
- Individuals manipulate threats for personal gain, rather than to enhance group welfare (Studies 1–2).

We used a three-person public goods game, which are common in economics [49]–[52], biology [16], [32]; psychology [15], [53], sociology [34], and other disciplines [54]. We based our public goods game on “tug-of-war” models of cooperation [18], [21], in which one group member occupied a high-resource, contestable position. In this game, cooperation increased group earnings and success but decreased one’s individual earnings and likelihood of achieving this privileged position. While abstract, such games model the incentive structure of many real-world situations in which individual and collective interests are in tension. Manipulation in our experiment can function as model for many types of real-world manipulation, such as exaggerating the threat of natural disasters or intergroup conflict. To avoid possible confusion, we clarify the goals of the paper and our operational definition of rank.

### Testing the Structural Factors, not the Underlying Psychology

Our goal is to examine structural factors that lead people to manipulate group threats. We are not testing the specific proximate psychological mechanisms underlying such manipulation. These psychological causes could include rational utility maximization, power-motivated approach tendencies, and implicit or explicit goals of holding rank or power. We are agnostic about the underlying psychology. Instead, we seek to examine circumstances under which threat manipulation arises, as well as the benefits that accrue for doing so. These questions are important to answer in order to understand the circumstances in which such psychologies will be activated outside the laboratory. Thus our focus is on behavior and its functional and situational causes, not psychological processes per se.

### Differing Definitions of “Rank”

The concept of “social rank” is widely used in the social sciences. However, the specific terminology varies widely within and across disciplines, and many types of social rank have been examined in prior research. To avoid confusion, we specify the type of rank that we investigate in the present paper. In doing so, we recognize that some disciplines use different terms, and we invite researchers who disagree with our operational definition to substitute their preferred term for the concept we are studying.

In studies 1 and 2, we define privilege or high rank as a contestable position that offers greater material resources than low-ranking positions. Despite the variability in terms and conceptions, access to resources and contestability are two commonly used and theoretically relevant dimensions of social hierarchies [20]. Those in privileged positions sometimes also have behavioral options that others do not (e.g. the ability to control others’ fates or earnings, [47], [55]), though these are not necessary features of such positions [27], [55]–[57]. We examine privileged positions both without (Studies 1–2) and with (Study 3) control over others’ earnings.

Because our conception of privileged positions (in Studies 1 and 2) is based on only two features – dominance-based contestability and higher wealth – it serves as a fairly minimal definition of a privileged position; more minimal definitions of “privileged positions” are possible (e.g. calling someone the “leader” without granting any actual privileges, [58]), but this is beyond our current focus on materially valuable positions. As such, our design in Study 1 provides a conservative test of the hypothesis that those in privileged positions will spend more on manipulating group threats. Study 3 broadens the definition of privilege to include control over outcomes. Once again, we test the structural aspects of threat manipulation, not the underlying psychological processes.

We focus here solely on positions that are gained and lost through dominance contests or resource-based power struggles. We do not examine positions that are gained through prestige [27], [59], a reputation for cooperative behavior [60]–[63], or personal charisma [64]. The latter routes to high rank likely operate through different mechanisms than the dominance contests we examine here, and are left for future work.

Dominance contests and resource-based power struggles are won by those with the greatest ability to invest in the competition, so investing in the entire group will decrease one’s relative ability to compete for rank [21], [27], [65]. For example, a fundamental dilemma faced by despots is that any resources they distribute to the military or other elites may in turn be used to overthrow them [24]. A key to dictators’ long-term survival is therefore forestalling a coup d’etat from a rival coalition of elites [66]. Perhaps as a result, the “diversionary” use of military force abroad to shore up political support at home occurs when a despot’s grip on power is weak, but not when it is strong [67]. Other real-world examples with similar tradeoffs include contests for gang leadership; competition over rankings based on relative wealth within society; non-human dominance hierarchies; investing in civil war versus another country (e.g. Chinese Communists and Kuomintang versus Japanese in War World II); or sometimes even candidates for leadership of a political party using advertisements that benefit the whole party versus attack their opponent.

Similarly, real-life groups may vary in whether all members or only some members can manipulate group threats. For example, within political parties, all candidates for a nomination can suppress “mudslinging” against themselves, by claiming that such practices may lose the party the general election (an external threat). In repressive regimes, in contrast, information may be so tightly controlled that only the leaders can manufacture or exaggerate threats. In our studies, we allow all individuals to manipulate the threat; logically, the only way to determine whether rank increases manipulation is to allow all ranks to manipulate others. By giving everyone the option to manipulate others, it provides a direct test of the idea that those in privileged positions are more likely to manipulate threats. It also tests whether possessing such a position *increases* an individual’s likelihood of investing in manipulation.

## Study 1

### Methods

#### Participants

Participants were 31 males and 35 females from the Cornell University community between 18 and 55 years old (mean age 21.9 years ± SD 6.6 years, median age 20 years) recruited via posters, announcements in class, e-mails, and from a website for experiments at Cornell. Each session included two separate groups of three participants. All participants gave written consent before participating. Data were anonymized, in that no personally identifying information about participants was associated with the data. These methods were approved by the Institutional Review Boards at Cornell University (studies 1 & 2), Indiana University (Study 3), and the University of Guelph.

#### Payment

During the experiment, participants earned “lab dollars” (henceforth L$), based on their decisions and the decisions of their group members. L$ were exchangeable to US dollars after the study at the pre-announced rate of 10∶1; participants were paid based on three randomly-selected rounds plus a $5 show-up payment (later $7). Earnings averaged $14.45 (± s.d. $5.25).

#### Anonymity

Partitions prevented visual contact between participants, and communication was not permitted. All decisions were made anonymously via computers; the study was programmed and conducted with the software z-Tree [68]. No deception was used: all participants received full instructions (see text S1 for screenshots) and passed a comprehension test before participating.

#### Experimental task: modified “public goods game”

Participants completed 20 rounds of a three-person “public goods game”. Each round, participants received an endowment (L$50 or L$80, described below), and could contribute any amount of this towards a group fund. All contributions to the group fund were summed, multiplied by 1.5, and divided equally among all group members regardless of what each person contributed. Thus, contributions are collectively beneficial because they increase the total amount of resources available to the group by 50%, but are personally costly because each individual receives only L$0.50 for each L$1 he/she contributes. The dilemma is that individuals always fare better by not contributing, but if all follow this strategy, they fare worse than if all had contributed.

**Figure 1:**
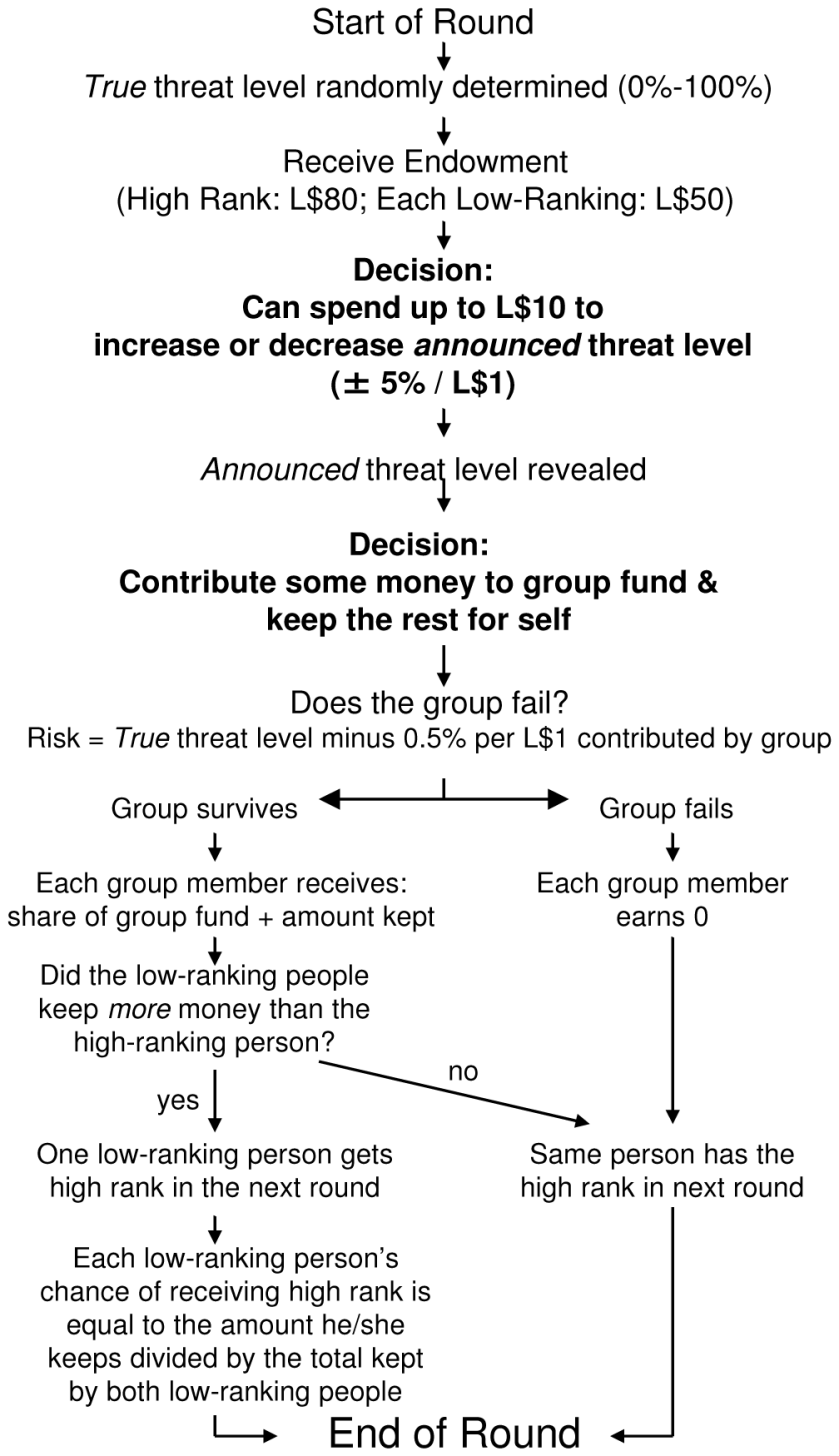
Flowchart of each of the 20 rounds of the study from the perspective of each participant. Participants' decisions are in bold.

To test the idea that leaders will manipulate others’ perceptions of group threats, we extended the standard public goods setting in two ways. First, we allowed for the possibility of a “group failure” where all participants earned zero in a given round (and for the previous) if contributions failed to surmount an external threat. We then allowed participants to pay to raise or lower what others saw as the risk of this “failure” occurring. Second, we granted privileged positions to some participants – L$80 at the start of each round instead of L$50– and made these privileged positions contestable based on investments in within-group competition. These extensions advance the paradigm of public goods games to allow for their use in analyzing a broader range of situations, including the presence of mutual threats, intragroup stratification, and the capacity for threat manipulation. Figure 1 shows the order of events in each of the 20 rounds.

#### Threat level and manipulation

At the start of each round, the computer randomly determined a risk of failure for the group (the “threat level”: 0%–100%, Figure 1). If the group failed in a given round, all members would earn zero for that round and the previous, losing both their share of the public good *and* any remaining private endowment. This simulates destruction of group resources at the hands of hostile out-groups [14], loss of food due to overuse of natural resources [28], or failure to respond effectively to natural disasters like hurricanes, floods, or forest fires. This risk of group failure could be reduced by contributions to the group fund: each L$1 contributed reduced the risk of group failure by 0.5%. For example, if the computer-determined threat level was 50% and the group members contributed a total of L$80, then at the end of the round there would be a 10% chance (i.e. 50% - (80*0.5%)) that everyone would earn zero for that round and the previous. Thus, in addition to increasing the value of the public good, contributions also increased the group’s likelihood of success.

In a novel extension of prior public goods games, we also allowed participants to invest resources in manipulating the apparent threat level. The *true* (i.e. unmanipulated) threat level was not known to participants in any round. Instead, the *announced* threat could be raised and lowered by participants who were willing to pay to do so. Participants could invest up to L$10 to increase or decrease the threat level that was announced to everyone (the “*announced* threat level”). Each L$1 spent changed the announced threat level by 5%, but did not affect the *true* risk of group failure. For example, if the *true* threat level was 50%, and one participant paid L$5 to increase the threat level, then the *announced* threat level would be 75% (50%+(5%*5)) but the actual risk of group failure would still be 50% (minus any contributions to the group fund). Thus, the sole purpose of changing the *announced* threat level was to increase or decrease others’ perceptions about the group’s risk of failure. The *announced* threat level was presented before participants decided how much to contribute towards the public good. The instructions used the neutral terms “increasing or decreasing the announced threat level” rather than the term “manipulation”.

#### Privileged positions and competition

In an additional extension of the standard public goods setting, we stratified the groups into “high-ranking” and “low-ranking” positions. This position was randomly assigned in the first round, after which we allowed participants to compete for the privileged position. Within each three-person group, one member (the “high-ranking participant”) was granted the privileged position of receiving an L$80 endowment each round whereas the other two members received endowments of L$50 (the “low-ranking participants”) (for other work on asymmetric endowments, see for example [69]–[72]).

In many real groups, competing for within-group position is costly, requiring time, effort, and resources. Because we focus on hierarchies that resemble dominance contests based on raw competitive power [18], [27], the privileged positions in Study 1 were contestable based on relative investments in within-group competition: those who invest more in such competition have a greater chance of winning the privileged position. We used relative amounts kept (i.e. amounts not spent on contribution or manipulation) as our measure of investment in within-group competition. This is because cooperating with the group and competing for rank are mutually exclusive activities: to the extent that one invests resources in helping the group, those resources are not available for investment in within-group competitions for dominance-based rank. Additionally, failure to cooperate is often seen as “competition” because it increases the non-cooperator’s payoff relative to cooperators [18], [73]–[74]. Thus, in our study, participants could increase their chances of attaining the high-ranking position by contributing less to the group; this represents the trade-off between cooperating with the group and competing for within-group position.

We instituted within-group dominance competition as follows. The high-ranking group member lost his/her position if the low-ranking members kept more money combined than did the high-ranking person, i.e. a “revolutionary coalition” [41] could overthrow the high-ranking individual. If such a supplanting occurred, each low-ranking person’s probability of attaining the high rank depended on the amount kept relative to the other low-ranking person. For example, if A (the high-ranking person) kept L$2 when B kept L$1 and C kept L$2, then A would be supplanted because he/she kept less than B and C combined (L$3). B would then have a 1/3 chance of attaining high rank for the following round whereas C would have a 2/3 chance. To avoid giving low-ranking members the perverse incentive of causing a group failure in order to effect a change in rank, supplantings only occurred in rounds where the group succeeded, i.e. if the group failed in one round, the positions stayed the same for the next round.

#### Summary of each round

Figure 1 shows the order of events in each of the 20 rounds. First, the computer randomly determined a *true* threat level (0%–100%). Participants then received their L$50 or L$80 endowments and decided how much to spend on increasing or decreasing the *announced* threat level. The *announced* threat level was equal to the *true* threat level plus or minus any increases or decreases that participants paid for (±5% per L$1 spent on manipulation). After seeing the *announced* threat level, participants decided how much to contribute to the group fund, and how much to keep.

The probability of group failure was equal to the *true* threat level minus 0.5% for each L$1 contributed to the group fund. If the group failed, then everyone earned zero for that round and the previous round, and everyone kept the same rank for the following round. If the group succeeded, then each person’s earnings were equal to the amount he/she kept plus 0.5 times the total group contributions, and a supplanting could occur. If the high-ranking person kept more money than the two low-ranking persons combined, then the former retained his/her position for the following round. If the two low-ranking persons kept more money in total than the high-ranking individual, then one of the low-ranking individuals would receive high rank for the following round. In this case, each low-ranking person’s chance of achieving high rank was equal to his/her amount kept divided by the total amount kept by both low-ranking persons. Participants did not know the total number of rounds.

#### Practice rounds

Participants completed five practice rounds to familiarize themselves with the procedure. These rounds were identical to the non-practice rounds except that they did not count towards participants’ study pay. After the practice rounds concluded, the privileged position was randomly re-assigned for the first round of the non-practice rounds. We took this step to eliminate any influence of the practice rounds on the non-practice rounds. In all subsequent rounds, participants earned or lost high rank as described above.

### Results & Discussion

#### Analytical strategy

The unit of analysis is one decision by one participant on a given round of the study. As interdependence within individuals and groups violates the assumptions of standard ANOVA or regression approaches [75], we employ a multilevel model in which rounds are nested within participants and participants are nested within groups, using the GLLAMM program in STATA [76]. We use a random intercepts model that assumes that baseline levels of behavior vary across levels, and effects of the predictor variables remain constant. There were two relevant dependent variables: net percent of endowment spent on increasing the threat level (i.e. amounts spent to increase threats minus amounts spent to decrease them) and percent of endowment contributed to the group fund (which is always equal or greater than zero); these two variables were analyzed separately. All presented *p*-values are two-tailed.

#### Manipulating threats

Although participants could increase or decrease the apparent threat level, they overwhelmingly chose to increase it rather than decrease it: the net outcome was that high-ranking participants increased the threat level on average in 20/20 rounds (binomial *p*<.005) and low-ranking participants did so in 18/20 rounds (binomial *p*<.0005). Text S2 gives the descriptive statistics in Studies 1–3.

**Figure 2:**
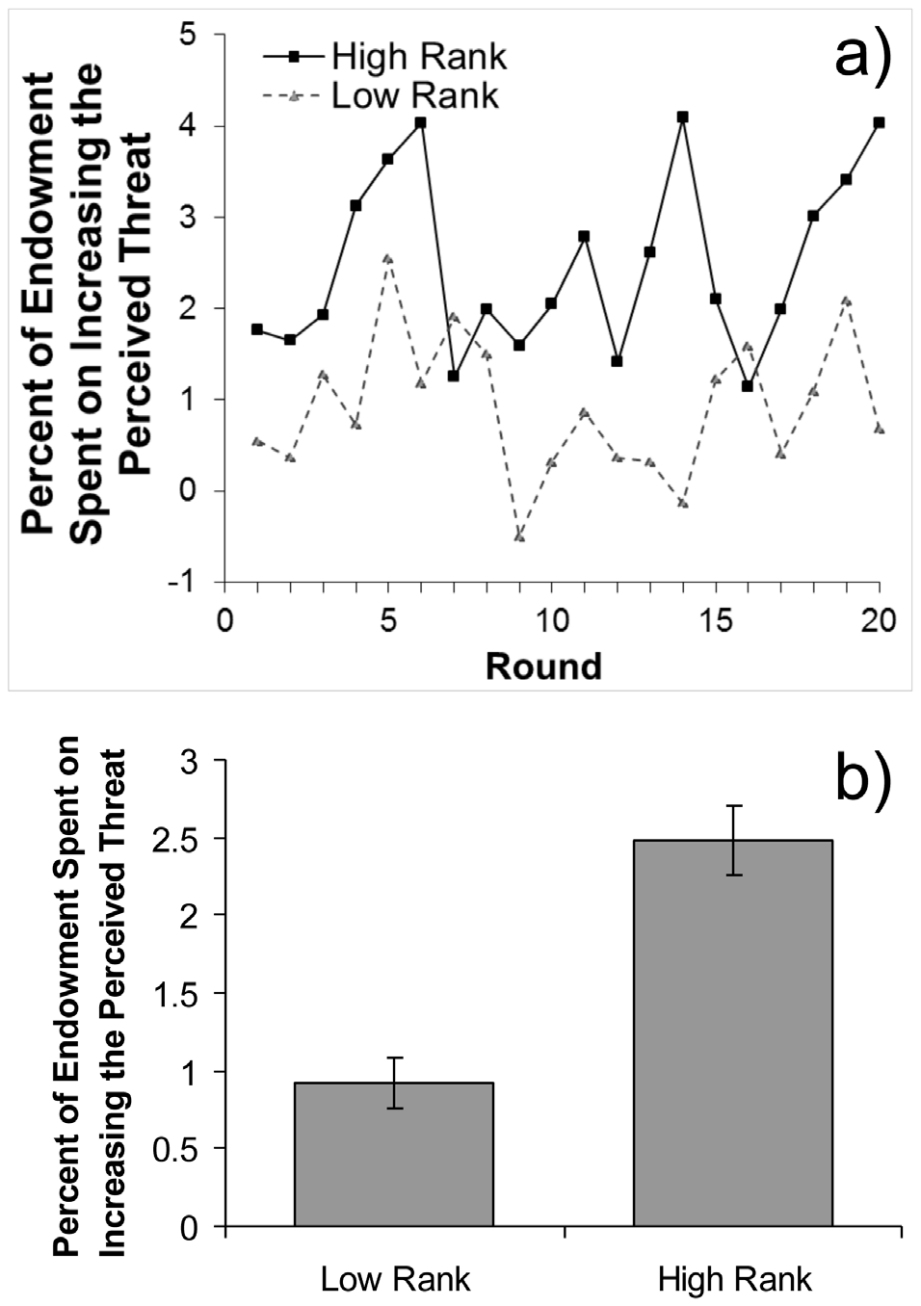
Average manipulation in Study 1. (a) Percentage of endowments (and standard error of the means) spent by high-ranking participants (solid line) and low-ranking participants (dashed line) on *increasing* the perceived threat level across rounds and (b) this manipulation overall. Positive (negative) numbers represent paying to raise (decrease) perceived threat levels. High- and low-ranking participants both paid to raise threat levels on average (both *p*s <.005), but high-ranking participants did so more than did low-ranking participants (*b* = 0.98, *z* = 3.63, *p*<.001).

As predicted, high-ranking participants spent a greater percentage of their endowment on increasing the apparent threat than did low-ranking participants (18/20 rounds, binomial *p*<.005, Figure 2a); the former spent an average of 2.5% of their endowments on net increases in the apparent threat level (i.e. L$2.0, or 1/5 of the maximum possible of L$10) whereas the latter spent an average of 0.9% (L$0.45), Figure 2b. This effect of rank was significant even after controlling for the effect of rounds (*b* = 0.98, *z* = 3.63, *p*<.001, Table 1). These analyses support our predictions that participants will pay to increase rather than decrease apparent threats, and will especially do so when possessing high rank.

**Table 1:** Effects on independent variables on manipulation and contributions in Study 1.

Fixed Effects	Manipulation	Contribution
Rank^a^	0.98** (0.27)	−3.70* (1.68)
Perceived Threat^b^		0.31** (0.02)
Round^c^	−0.01 (0.02)	−0.37** (0.13)
Constant	1.20** (0.39)	19.74** (3.62)
Random Effects		
Individual-level random errors	2.34** (0.20)	13.5** (1.7)
Group-level random errors	0.00 (0.60)	12.30** (2.80)

a 0 = low rank, 1 = high rank.

b Perceived threats are not included in the manipulations analysis because participants made decisions about manipulating perceived threats *before* threat levels were announced.

c There were 20 rounds.

Multilevel model of percents of endowment (and standard errors of the coefficients) spent on increasing the threat level (column 1) and contributing to the group (column 2) in Study 1. Numbers represent the effect of a one unit change in the independent variables on the percent of endowment spent on manipulation or contribution. Each model is based on 1320 observations (66 participants across 20 rounds).

* *p*<.05;

** *p*<.01.

#### Contributions to the group fund

**Figure 3:**
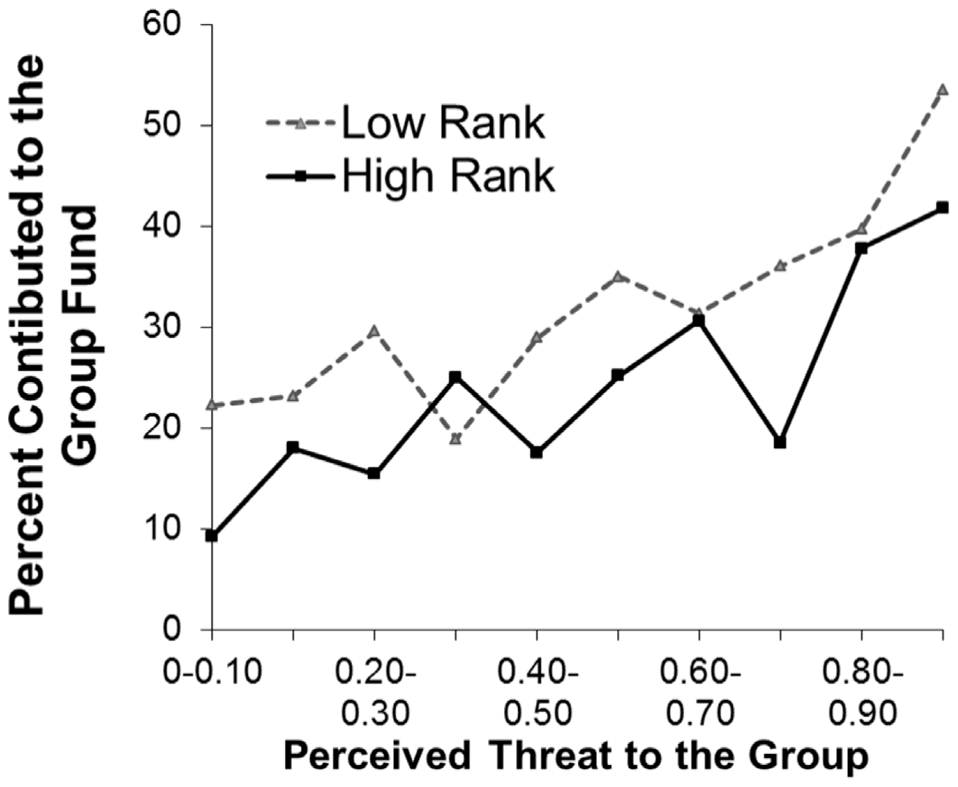
Average percentage of endowments contributed to the group by high- and low-ranking participants in Study 1 at different perceived probabilities of group failure. Contributions increased as perceived threats increased (*b* = 0.31, *z* = 15.5, *p*<.001). High-ranking participants contributed a lower percent of their endowment than did low-ranking participants (*b* = −3.70, *z* = −2.20, *p* = .028).

This manipulation of perceived threats was effective: on average, each 1% increase in the perceived threat level from a participant’s perspective (i.e. after subtracting his/her own manipulation) resulted in participants contributing an extra 0.31% of their endowment to the group fund (*b* = .31, *z* = 15.50, *p*<.001, Figure 3, Table 1). This demonstrates that people do show “threat-dependent” cooperation by contributing more when group threats are high. Ancillary analyses found that high- and low-ranking group members raised their proportional contributions similarly in response to threats (interaction of position and perceived threat: *z* = −0.54, *p* = .59).

Although not related to our central hypotheses, we present two other effects on contributions to the group fund. First, and consistent with previous research using Voluntary Contribution Mechanisms [50], contributions declined across rounds as participants gained experience in the task (*b* = −0.37, *z* = −2.85, *p* = .004, text S3). Second, those with high rank contributed a smaller percentage of their endowment than did those with low rank (*b* = −3.70, *z* = −2.20, *p* = .028, Figure 3, Table 1) despite contributing more in actual lab dollars (*b* = 5.29, *z* = 5.41, *p*<.001; results not shown). This pattern is generally consistent with past economic experiments on heterogeneous endowments, in that high-endowment participants contribute equal to or more in absolute terms (probably because they simply have more to contribute), but tend to contribute less in proportional terms or relative to Nash equilibrium predictions than low endowment participants do([69]–[70], [77]–[78], but see [79]).

On average, groups failed on 21% of the rounds. Low-ranking participants supplanted the high-ranking person in 60.1% of the rounds where the group succeeded (i.e. 47.5% of all rounds). Group failure had no effects on contributions or manipulation in the subsequent round, nor was there an interaction of past failure with rank (see text S4).

#### Cost-Effectiveness of manipulation

The manipulation by high-ranking participants alone caused a 10% increase in perceived threats *each round* (i.e. L$2 spent on manipulation on average, multiplied by 5%/L$), which resulted in each of the two low-ranking participants contributing an estimated extra 3.1% of their L$50 endowments (i.e. L$1.55 each or L$3.1 total each round). Thus, manipulation was a cost-effective way to increase group members’ contributions and reduce their ability to compete for within-group position.

#### Is manipulation prosocial

We argue that high-ranking participants manipulate the threat level to increase others’ contributions and reduce their ability to compete over rank. However, an alternative interpretation is that such manipulation is actually prosocial behavior (“benevolent lies”) intended to motivate cooperation and help the group succeed. This alternative explanation is unlikely for at least three reasons, which we will describe only briefly in the interest of space. Firstly, ancillary analyses (available in text S5) find that the amount spent on manipulation does not significantly increase efficiency at the group level. The benefits of manipulation thus do not significantly outweigh the costs at the group level, even accounting for the costs of group failure. Secondly, a mathematical argument: it is the low-ranking participants who pay the higher contributions when the high-ranking player manipulates the threat level, and this extra cost exactly cancels out their private gains, and must do so mathematically, because each participant receives L$0.50 for each extra dollar contributed by each of the two low-ranking persons (including him/herself). A truly prosocial leader would simply donate money to the public good rather than spend it on manipulation, as that would both directly increase the payoffs of other group members and indirectly increase their contributions in future rounds because of conditional cooperation (i.e., the tendency to reciprocate cooperation, see [80]–[81]). As such, manipulation seems inefficient as a means of improving group welfare, but efficient as a means of decreasing others’ competitive ability. Thirdly, the data show that manipulation is more characteristic of low than high contributors. Low contributors perform more manipulation: above-median contributors spend marginally less on manipulation than below mean contributors (effect of being a high contributor: *b* = −1.05, *z* = −1.69, two-tailed *p*<0.10, full analysis available upon request). This shows that low, not high, contributors disproportionately manipulate group threats. Taken together these analyses strongly suggest that threat manipulation is not a “benevolent lie” whose principal function is to benefit the group.

#### Summary of results

The results of Study 1 strongly support the hypotheses. Participants paid to increase apparent threats to the group, and as predicted, those holding privileged positions invested a greater percentage of their endowment in manipulation. The manipulation of perceived threats also produced a net advantage in the contest over within-group position. Furthermore, this manipulation occurred despite the risk to the group, and was performed more by less cooperative people.

## Study 2

We hypothesized that the manipulation of group threats by individuals in privileged positions is driven by *competition* over position within groups, and Study 1′s results are consistent with this argument. Supporting this, Maner and Mead [47] found that leaders were more likely to behave selfishly when their positions were unstable than when they were secure. An alternative explanation is that simply *occupying* a privileged position encourages threat manipulation. Recent work has shown, for example, that holding a privileged position within a group increases people’s disinhibition [43], objectification of others [44], and decreases perspective-taking [45]. These tendencies alone could increase individuals’ willingness to manipulate apparent threats to the group. Although these explanatory mechanisms – privilege and competition for privilege – are not mutually exclusive, it is important to determine whether one or both underlie threat manipulation.

To distinguish between these two possible mechanisms, Study 2 manipulates whether participants compete for the privileged positions, as in Study 1, or whether these positions are randomly assigned.

### Methods

Ninety-six new participants from the same population (39 males and 57 females, aged 18–67 years, mean age 21.8± s.d. 6.7 years, median age 21 years) did the same cooperative game from Study 1 (i.e. 32 groups), but in two experimental conditions in a within-subject design. The Contestable Rank condition was identical to Study 1, in which the possession of the privileged position was based on relative amounts kept. The Random Rank condition was similar except that the privileged position was randomly re-assigned to a low-ranking participant in 60.1% of rounds in which the group succeeded (to match the frequency of supplantings when groups succeeded in Study 1). Groups interacted for 20 rounds in each experimental condition, with order counterbalanced between groups. The order of conditions did not significantly affect the results, and so order effects are not included in the models. In the statistical model (Table 2), Rank and Contestability are binary variables, with Low Rank and the Random Rank condition, respectively, as the reference categories. Thus, the main effect of Rank in the model indicates the effect of rank in the Random Rank condition, net of other control variables. Similarly, the main effect of Contestability indicates the effect of contestability for low-ranking participants. The Rank×Contestability interaction indicates whether the contestability manipulation significantly increases the effect of rank.

**Table 2:** Effects of independent variables on manipulation and contributions in Study 2.

Fixed Effects	Manipulation	Contribution
Rank^a^	0.41* (0.20)	−7.00** (1.29)
Contestability^b^	−0.67** (0.16)	−12.40** (1.05)
Perceived Threat^c^		0.34** (0.01)
Round^d^	0.01* (0.005)	−0.14** (0.04)
Rank×Contestability	1.25** (0.28)	0.01 (1.87)
Constant	0.95* (0.30)	35.53** (3.59)
Random Effects		
Individual-level random errors	2.50** (0.20)	15.2** (1.4)
Group-level random errors	−0.00 (0.60)	16.69** (2.70)

a 0 = low rank, 1 = high rank.

b 0 = random rank, 1 = contestable rank.

c Perceived threats are not included in the manipulations analysis because participants made decisions about manipulating perceived threats *before* threat levels were announced.

d There were 40 rounds (20 with contestable rank and 20 with random rank with order counterbalanced).

Multilevel model of percents of endowment (and standard errors of the coefficients) spent on increasing the threat level (column 1) and contributing to the group (column 2) in Study 2. Numbers represent the effect of a one unit change in the independent variables on the percent of endowment spent on manipulation or contribution. Each model is based on 3840 observations (96 participants across 40 rounds).

* *p*<.05;

** *p*<.01.

### Results & Discussion

**Figure 4:**
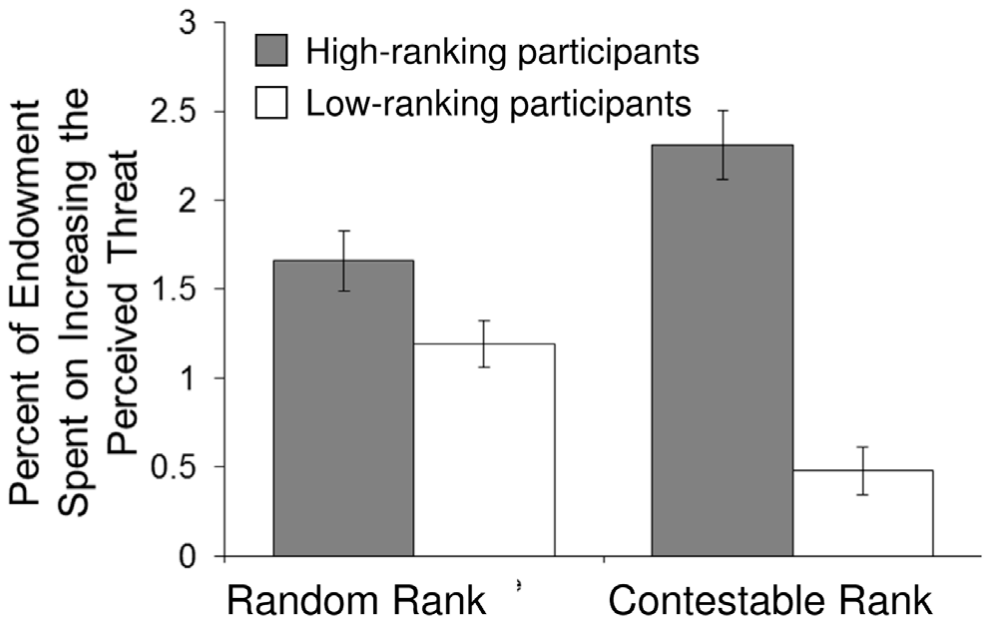
Average percent of endowment (and standard error of the means) spent on *increasing* the perceived threat level, Study 2.

In the Random Rank condition, high-ranking participants spent a higher percentage of their endowment to increase the threat levels than did low-ranking participants (1.7% vs. 1.2% or L$1.33 vs. L$0.60, respectively, Figure 4), and the main effect of Rank shows that this difference is significant (b = 0.41, z = 2.05, p<0.05, Table 2). This shows that mere *possession* of a privileged position is sufficient to cause differences in manipulating threats.

As predicted, *competition* over rank magnifies this effect in two ways. First, low-ranking participants spend *less* on increasing the threat level in the Contestable Rank condition (0.5% of endowment, or L$0.24) than in the Random Rank condition, as shown by the significant negative main effect of Contestability on threat manipulation (b = −0.67, z = 4.19, p<0.01). Second, high-ranking participants spend more on increasing the apparent threat when individuals competed over rank than when rank was randomly assigned (Contestable Rank condition: 2.3% of endowment, or L$1.85; Random Rank condition: 1.7% or L$1.33). This latter effect is demonstrated by the presence of a significant, positive interaction of Rank×Contestability (b = 1.25, z = 4.46, p<0.01). This interaction effect is greater than the main effect of contestability, indicating that the contestability of rank causes high-ranking individuals to spend more on increasing the apparent threat (1.25–0.67 = 0.58). As further support for this analysis, we examined high and low rank separately: contestability has a significant, positive main effect on manipulation by high-ranking participants (b = 0.53, z = 2.70, p<0.01), and a significant, negative main effect on manipulation by low-ranking participants (b = −0.64, z = −3.88, p<0.0005). Overall, consistent with our predictions, contestability tends to increase the amount that high-ranking individuals invest in manipulation, while decreasing the amount invested by low-ranking individuals.

The contestability of rank had two primary effects on contributions to the group fund. Not surprisingly, contributions were lower when rank was contestable (50% vs. 38%, t = −4.80, p<0.0001, paired *t*-test of group-level means). As a result, groups failed more often on average in the Contestable Rank condition than in the Random Rank condition (24% vs. 15%, t = 4.83, p<0.005, paired *t*-test).

Study 2 also replicated five other findings from Study 1. First, manipulation of perceived threats was effective, in that each 1% increase in the perceived threat level (from a participant’s perspective) resulted in the low-ranking participants contributing an extra 0.34% of their endowment to the group fund (*b* = .34, *z* = 34.00 *p*< <0.001, Table 2). Second, this manipulation was cost-effective, in that the L$1.98 that high-ranking participants spend each round on manipulation (across both conditions) results in a 9.9% increase in the perceived threat (i.e. L$1.98×5% increase in the threat level per L$). On average, this results in each low-ranking participant contributing (9.9×0.34) = 3.37% more of their L$50 endowment (i.e. L$1.685 each, L$3.37 total). Third, high-ranking participants contributed a smaller percentage of their endowments than did low-ranking participants, as shown by the negative main effect of rank on contribution (*b* = −7.00, *z* = 5.43, *p*<0.01, Table 2, column 2); this did not interact with the contestability of rank (*b* = 0.01, *z* = 0.01, *p* = .992). Fourth, contributions declined over time (*b* = −0.14, *z* = −3.5, *p* = 0.01, text S3). Fifth, group failure had no effect on the proportions of endowment spent on manipulation or contributions in the subsequent round (see text S4).

#### Is manipulation prosocial?

As in Study 1, above-median contributors spend less on manipulation (*b* = −1.94, *z* = −3.94, *p*<0.0005), and among high-ranking participants, the amount spent on manipulation was negatively related to proportion of the remaining endowment contributed to the group fund (*b* = −0.77, *z* = −3.69, *p*<0.0005). Further evidence against manipulation being a “benevolent lie” is that high-ranking participants manipulated more when rank was contestable than when it was randomly assigned (see above). This pattern held even after controlling for the other group members’ previous contributions (contestability×rank effect with prior contribution controls: *b* = 1.23, *z* = 4.32; *p*<0.0005, available upon request), which suggests the increase in manipulation is not simply due to the need to make up for low contributions in that condition. Together, as in Study 1, these suggest that manipulation does not primarily function for the benefit of the group.

#### Summary of study 2 results

Study 2 replicates the main findings of Study 1. It also showed that *possession* of a privileged position is sufficient to cause differences in manipulation. Importantly, Study 2 provided insight into the situational factors at work by demonstrating that the effects of rank are magnified when individuals compete for rank. When rank is contestable, high-ranking participants increased their manipulation, whereas low-ranking participants reduced theirs – future studies could test whether this decrease is caused by low-ranking participants needing to cooperate to supplant the high-ranking participant (“revolutionary coalitions”, [41]). This contestability of rank also reduces group cooperation, and thus increases group failure. Our experimental manipulation, and the ancillary analyses, supports the hypothesis that concerns for rank lead high-ranking participants to manipulate the threat level.

## Study 3

Studies 1 and 2 both used a difference in endowment (L$50 vs. L$80) to operationalize the “privileged positions” held by high-ranking group members. This “thin” definition of rank demonstrates that a relatively minimal level of advantage can lead high-ranking individuals to manipulate apparent threats to the group. However, high-ranking individuals in both naturally occurring groups and laboratory studies sometimes enjoy other advantages over low-ranking individuals, especially direct control over the outcomes of low-ranking individuals (e.g. [47], [82]–[83]). In Study 3, we sought to replicate our main results using an expanded definition of rank that gave high-ranking members control over the outcomes of low-ranking group members. To do this, we added a condition where high-ranking members could divide the group’s earnings however they chose.

### Methods

One hundred eight new participants from Indiana University (43 males and 65 females, mean age 20.9± s.d. 3.28 years) did the same cooperative game from Study 1 (i.e. 36 groups), but in two experimental conditions in a within-subject design. The Baseline condition was identical to Study 1, in that the high- and low-ranking positions differed only in their endowments each round (L$80 vs. L$50). In the Extra Power condition, in addition to these differences in endowment, high-ranking participants were granted the power to distribute the group’s earnings: after the total group contributions were multiplied by 1.5 by the experimenter, the high-ranking member of each group could distribute this new total in any way he/she chose (compare this to the automatic equal division in the Baseline condition). Groups interacted for 20 rounds in each condition, with order counterbalanced between groups. Instead of being paid for a few randomly-selected rounds, participants were paid based on their average earnings across all rounds (average: US$26.97± s.d. US$18.90).

The order of conditions had no meaningful effect on the results (see text S6), so order effects are not included in the models below. In the statistical model (Table 3), Rank and Power are binary variables, with Low Rank and the Baseline condition, respectively, as the reference categories.

**Table 3:** Effects of independent variables on manipulation and contributions in Study 3.

Fixed Effects	Threat Manipulation no interaction	Threat Manipulation with interaction	Contribution with interaction
Rank^a^	0.35* (0.14)	0.13 (0.20)	−5.44** (1.41)
Extra Power Condition^b^	−0.34** (0.12)	−0.49** (0.16)	1.53 (1.11)
Perceived Threat^c^			0.20** (0.02)
Round^d^	0.01 (0.01)	0.01 (0.01)	−0.11** (0.04)
Rank×Extra Power		0.44 (0.28)	4.99* (1.97)
Constant	0.54+ (0.30)	0.61* (0.30)	33.64** (2.43)
Random Effects			
Individual-level random errors	2.68* (1.12)	2.68* (1.13)	16.94* (7.19)
Group-level random errors	−0.30 (1.30)	0.30 (1.40)	6.82 (6.26)

a 0 = low rank, 1 = high rank.

b 0 = Baseline condition, 1 = Extra Power condition.

c Perceived threats are not included in the manipulations analysis because participants made decisions about manipulating perceived threats *before* threat levels were announced.

d There were 40 rounds (20 in Baseline condition and 20 in Extra Power condition with order counterbalanced).

Multilevel model of percents of endowment (and standard errors of the coefficients) spent on increasing the threat level (columns 1–2) and contributing to the group (column 3) in Study 3. Numbers represent the effect of a one unit change in the independent variables on the percent of endowment spent on manipulation or contribution. Each model is based on 4,320 observations (108 participants across 40 rounds).

+*p*<0.10;

* *p*<.05;

** *p*<.01.

### Results & Discussion

**Figure 5:**
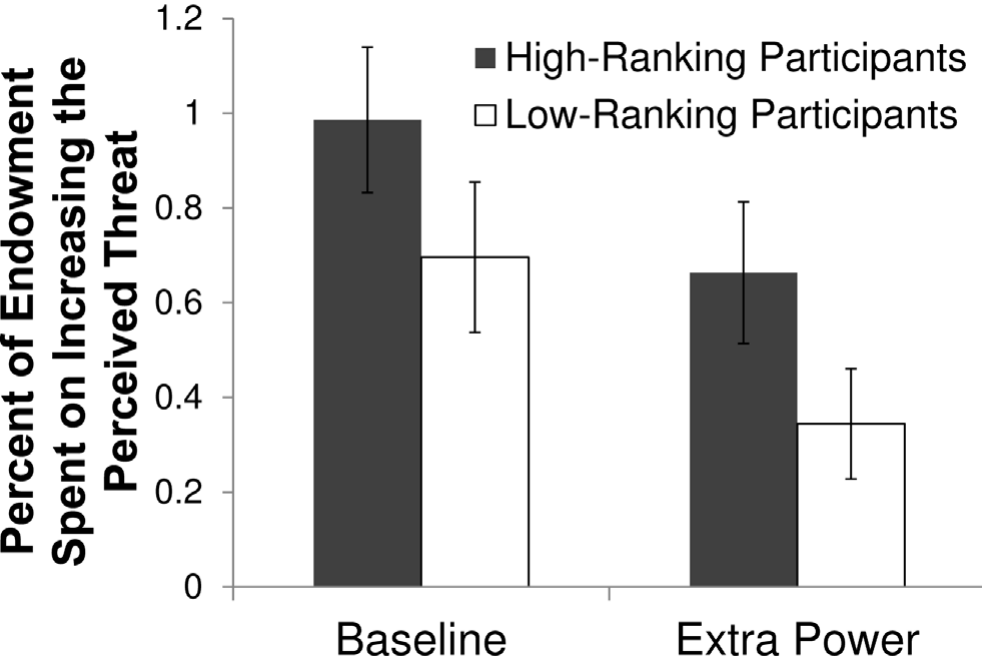
Average percent of endowment (and standard error of the means) spent on *increasing* the perceived threat level, Study 3.

We evaluate the results for the manipulation measure using a multilevel model that includes rank, the power manipulation, and a control for round as independent measures (Table 3). Consistent with studies 1 and 2, we find that high-ranking members invested a significantly greater percentage of their endowments in manipulating apparent group threats, compared to low-ranking members (Rank effect: *b* = 0.35, *z* = 2.40, *p* = .016, Table 3, model without interaction, see also Figure 5). In addition, participants invested significantly *lower* percentages of their endowments in manipulation when the high-ranking group member was more powerful (Extra Power effect: *b* = −0.34, *z* = −2.73, *p* = .006, Table 3, model without interaction, see also Figure 5). The effect of round was not significant, and substantively close to zero.

To assess whether the power manipulation qualifies the effect of rank, we added the Rank×Extra Power interaction effect to the analysis above. The power manipulation does not significantly qualify the effect of rank, as shown by the non-significant Rank×Extra Power interaction effect (*b* = 0.44, *z* = 1.60, *p* = .11). Although the Rank×Extra Power interaction is not significant, we investigate it more closely for two reasons. First, including this interaction attenuates the main effect of Rank, rendering it non-significant (*b* = 0.13, *z* = 0.64, *p* = .52). Second, the interaction effect is large enough to be substantively meaningful, and approaches (though does not reach) significance. For these reasons, we analyze the effect of rank separately by the Baseline and Extra Power conditions. These analyses find that high-ranking members spent proportionally more than low-ranking members to manipulate threats in the Extra Power condition (*b* = 0.40, *z* = 2.09, *p* = .036) but not the Baseline condition (*b* = 0.18, *z* = 0.78, *p* = .43). These findings suggest that if anything, the effects of rank are stronger – not weaker – when we expand our definition of “privileged position” to give greater power to the high-ranking members. As such, this indicates that effects of rank on threat manipulation are somewhat robust to changes in definitions of rank and privilege.

We next examine the effects of rank and power on contributions to the group. As in Studies 1–2, high-ranking members contributed a lower percentage of their endowment to the group fund than did low-ranking members. This is shown by the negative and significant main effect of rank in Table 3 (*b* = −5.44, *z* = −3.84, *p*<0.001). Low-ranking members contributed non-significantly different amounts to the group fund in the Baseline and Extra Power conditions (Rank effect: *b* = −1.53, *z* = −1.38, *p* = 0.166). The effect of rank was qualified by a positive and significant rank×power interaction, such that high-ranking members contributed more to the group fund in the Extra Power condition than in the Baseline condition (*b* = 4.99, *z* = 2.53, *p*<0.05).

We also replicate the findings from studies 1 and 2 that participants contribute less over time (Round effect: *b* = −0.11, *z* = −2.79, *p*<0.005, text S3), and contribute more at greater threat levels (Perceived threat effect: *b* = −0.20, *z* = −12.70, *p*<0.001). In terms of cost effectiveness in both conditions, each 1% increase in the perceived group threat was associated with a.2% increase in contribution. Because each L$1 spent results in a 5% increase in the threat level (see Methods), each $1 spent by the high-ranking participant results in each low-ranking participant contributing (5×0.20) = 1% more of their L$50 endowment (i.e. L$0.50 each, L$1.00 total). Thus, investing L$1 in manipulation results in a $1 increase in PG contributions, and also reduces the low-ranking individuals’ likelihood of winning the high ranking position in the next round.

We suspect that high-ranking group members contributed more in the Extra Power condition because contributing offered greater returns for them in that condition. This is due to the fact that high-ranking individuals could claim a larger share of the group’s productivity in that condition (see below). For example, a high-ranking individual who contributed their entire L$80 endowment to the group could claim up to the resulting L$120 in group production; in the baseline condition, the same level of contribution would net L$40. In fact, when high-ranking members could divide the group’s earnings (i.e., in the Extra Power condition), they gave themselves 57.1% of the earnings on average (± s.d. 27.7%), despite comprising only 37.1% of the group’s contributions on average (± s.d. 25.0). This disparity between equitable distributions and actual distributions was most pronounced when the high-ranking members contributed little (see text S7 for this and other analyses of distributions).

These unequal divisions may not have affected the contributions of low-ranking members if they felt that their lower shares were offset by the increased contributions from high-ranking members. As a result of the higher contribution levels, groups failed less often in the Extra Power condition than in the Baseline condition (16.7% vs. 23.5%, paired *t*_17_ = 3.13, *p* = .006).

#### Summary of study 3

High-ranking members spent more to increase the perceived threat than did low-ranking members, and if anything this was driven by the Extra Power condition (though the Rank×Power interaction was not significant). This suggests that our main result – that possessing a high-ranking position in the group leads individuals to exaggerate apparent threats – is robust to at least some changes in how we operationally define “high rank”. This helps us to generalize the result beyond the wealth-based definition of “privileged position” we used in Studies 1 and 2. Of course, it remains possible that there are yet other unstudied definitions of “rank” that would not produce our effects. Nevertheless, Study 3 should help alleviate some such concerns over definitions of “privilege” and “rank”.

Study 3 also found that high-ranking participants tended to contribute more when they were more powerful. However, their greater contributions in the Extra Power condition may be at least in part self-interested. High-ranking participants in the Extra Power condition tended to claim a disproportionate share of the public good, compared to their contribution to the public good.

## General Discussion

External threats, such as intergroup conflict or natural disasters, motivate group cohesion and self-sacrifice for the group [5], [8]–[9], [12], [30], [32], [34], [39]. We provide experimental evidence for an understudied “dark side” of this phenomenon, namely, people’s willingness to exploit others’ cooperation by manufacturing false threats to the group. We found that participants paid to exaggerate the apparent threat level, and that this tendency was greater among individuals who held a contestable high-resource position within the group. Furthermore, this manipulation was cost-effective for the high-ranking group members at reducing others’ ability to compete over rank. Study 2 demonstrated that mere possession of rank will elicit greater manipulation, but also that competition over rank exacerbates the tendency of high-ranking members to manipulate others. Study 3 demonstrates that our results generalize to a different definition of “privilege” which includes power over others’ outcomes.

By demonstrating that high-ranking group members manipulate others’ willingness to cooperate in the face of group threats, we extend other work showing that people are more willing to subordinate their group’s interest for their own when their leadership positions are unstable [47]. An additional way that our results contribute to this line of work is by showing that these strategies actually pay off in measurable terms, and as such we demonstrate why such strategies continue to exist. More generally, our results provide further insight into the tendency of individuals to behave in less prosocial ways when occupying high-ranking positions in a group [43]–[46]. To extend Lord Acton’s famous aphorism, it appears that “power corrupts, but competition for power corrupts absolutely”.

The presence of manipulation caused a sizeable increase in apparent threats. In non-laboratory groups, the cumulative effect of manipulation could be substantial, especially when the cost-effectiveness of manipulation is high. The percentages of endowments spent on manipulation in our studies do not translate directly into different currencies outside the laboratory (e.g. salaries, time, energy). However, the relative investment in manipulation by high-ranking members could be greater in systems where the differences in power, earnings, or reproduction are even greater between high and low rank, where manipulation is easier, or where high-ranking members can manipulate more than two group members simultaneously. Correspondingly, manipulation may be weaker in less stratified systems or as the costs of manipulation increase. Study 3 showed that different definitions of a privileged position can affect the levels of manipulation. Finally, increased levels of within-group competition will increase the incentives to manipulate others, but will also suppress responsiveness to group threats by decreasing cooperation (see “scale of competition”, [22]), and it is unknown which of these effects is greater.

In addition to increasing manipulation, competition over privileged positions resulted in lower cooperation. This is consistent with other recent work demonstrating the negative effects of within-group competition on levels of cooperation [22], [65]. Our results also give an example of the practical outcome of such competition: groups failed approximately 1.5 times more often when competition is present (15% vs. 24% of rounds). Competition over hierarchical positions can thus result in groups being less able to accomplish their goals, and insofar as this game is a model for groups dealing with external threats like natural disasters or hostile outgroups, within-group competition makes them less likely to succeed.

Manipulation probably cannot be used continuously, as multiple cries of wolf will cause audience skepticism [40]. For example, if union members notice that managers only warn of potential bankruptcy around the time of negotiations, then they may eventually disbelieve such warnings (and potentially suffer the consequences of bankruptcy if the threats turn out to be real). Manipulation is more likely when group members cannot easily or cheaply determine threat levels or detect deception, for example when anonymity or one-shot interactions prevent reputations for false alarms. Manipulation may also occur when leaders’ positions are unstable [39] such that they have little to lose from exposed manipulation, or when the cost to perceivers of missing an actual threat is high [40]. Eventually, a stable equilibrium should be reached with low levels of deception that will not completely undermine sensitivity to threats [84]; mathematical models are needed to determine such equilibrium points, and future work can investigate the conditions that evoke audience skepticism and counter-strategies. Threat manipulation and skepticism thereof, particularly in relation to contests over rank, underscores the complexity of group cooperation and the strategies that may promote or undermine it.

### Limitations and Future Research

Although the present study used university students, these effects of privilege on the manipulation of threats are predicted in any biological system where high-ranking group members disproportionately benefit from increased cooperation and/or decreased competition and where costly cooperation reduces one’s ability to compete (as in dominance contests). As such, manipulation of threat-dependent cooperation may represent an important component of social behavior. Examples of such systems include management-labor relations, human politics, dominance competitions, and reproductive skew within cooperatively breeding species. Non-humans animals create false threats to distract conspecifics [39], but whether they manipulate others’ threat-dependent cooperation remains to be investigated.

The present results are also limited because they used a single experimental setting. We used experimental economic games, which are designed as abstract settings that model a wide range of real-world situations with similar incentive structures. While this has the advantage of facilitating the systematic replication of our findings across studies one through three, it does not allow us to test the robustness of our findings with other operationalizations of the key variables. We encourage future studies on threat manipulation using different methods and settings.

Our studies raise many questions for future research. For example, it is currently unknown whether this manipulation will be more pronounced or more effective when the threats represent outgroups instead of impersonal threats. It is also unknown how the presence of information about manipulation will affect people’s responsiveness to threats (and thus the payoffs for using manipulation). The cognitive underpinnings require investigation, particularly as they relate to perceptions of competition, because removing competition reduced the manipulation by privileged members. Finally, these effects must be tested in other types of hierarchies where there is no trade-off between helping the group and competing for rank, such as democracies instead of dominance struggles. Here we focus on establishing the basic effect and its causes. Our method provides a way of analyzing human behavior in these situations, and thus our studies advance the experimental paradigm of cooperative games by providing a means of incorporating mutual threats and manipulation.

## Supporting Information

Text S1 **Screenshots of the instructions for the experiment.**
(DOCX)

Text S2 **Descriptive statistics of the average manipulation and contributions in Studies 1–3.**
(DOCX)

Text S3 **Average contributions to the group fund across rounds.** (a) Study 1. (b) Study 2. (c) Study 3.
(DOCX)

Text S4 **Effects of group failure on manipulation and contributions in Studies 1–3.**
(DOCX)

Text S5 **Effect of manipulation on earnings efficiency in Studies 1–3.**
(DOCX)

Text S6 **Detailed analysis of the division of the public good by the high-ranking player in Study 3.**
(DOCX)

Text S7 **Analysis of order effects in Studies 2–3.**
(DOCX)

Dataset S1 **Raw data for Study 1.**
(CSV)

Dataset S2 **Raw data for Study 2.**
(CSV)

Dataset S3 **Raw data for Study 3.**
(CSV)

Protocol S1 **DO-file of data analysis used in STATA.**
(DO) Click here for additional data file.
