# Supplementary material for: Who Cries Wolf, and When? Manipulation of Perceived Threats to Preserve Rank in Cooperative Groups
Source: PLoS One. 2013 Sep 12;8(9):e73863. doi: 10.1371/journal.pone.0073863 (PMC3772075; doi:10.1371/journal.pone.0073863)

Supporting Text S1: Screenshots of Instructions

The following are screenshots from the instructions program for the task used in the experiment. Within each page, participants had to click a button after each paragraph to proceed to the next paragraph, whereupon the focus (darker font) shifted to the new paragraph; the following screenshots represent the complete pages from the instructions.


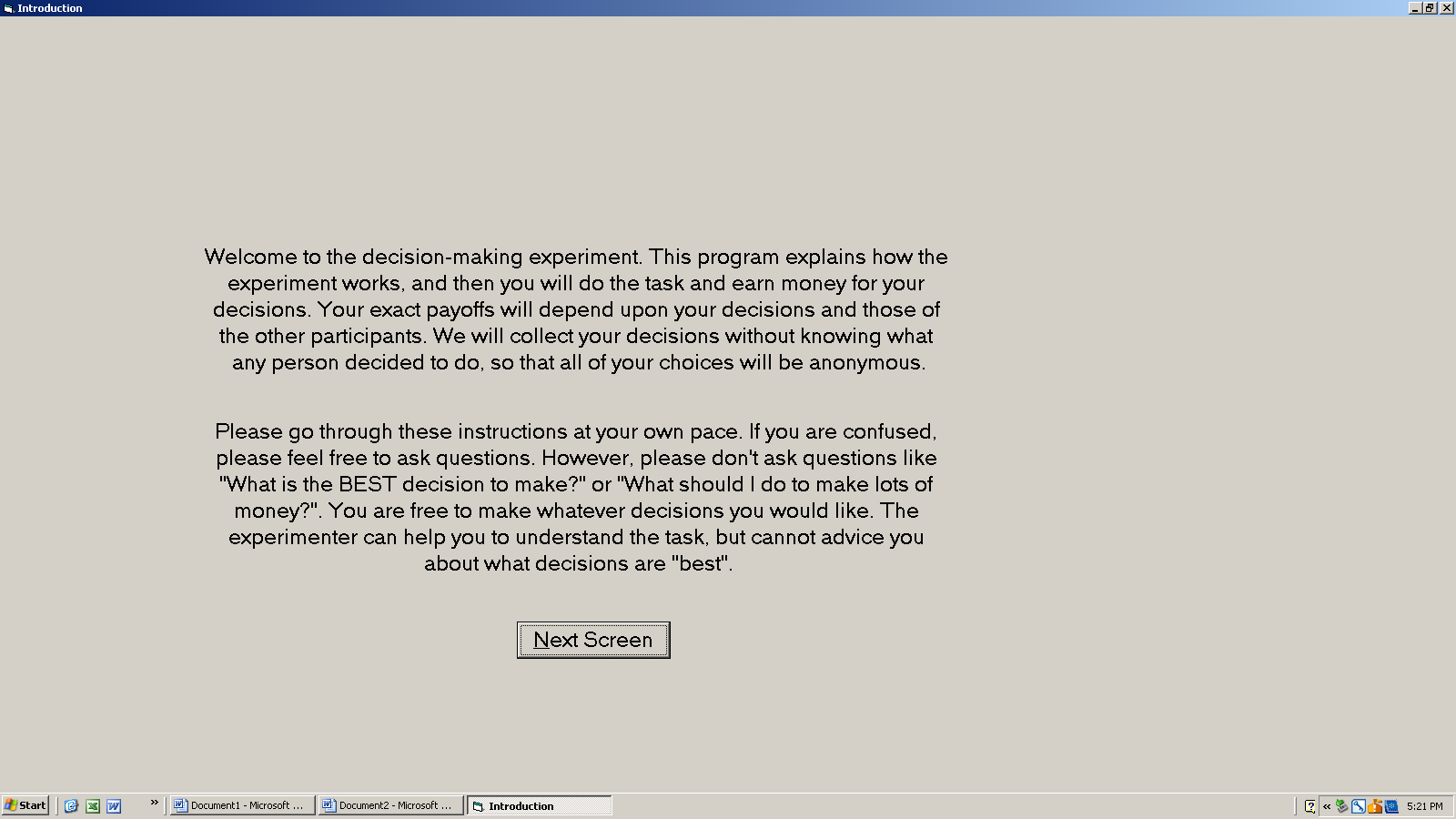


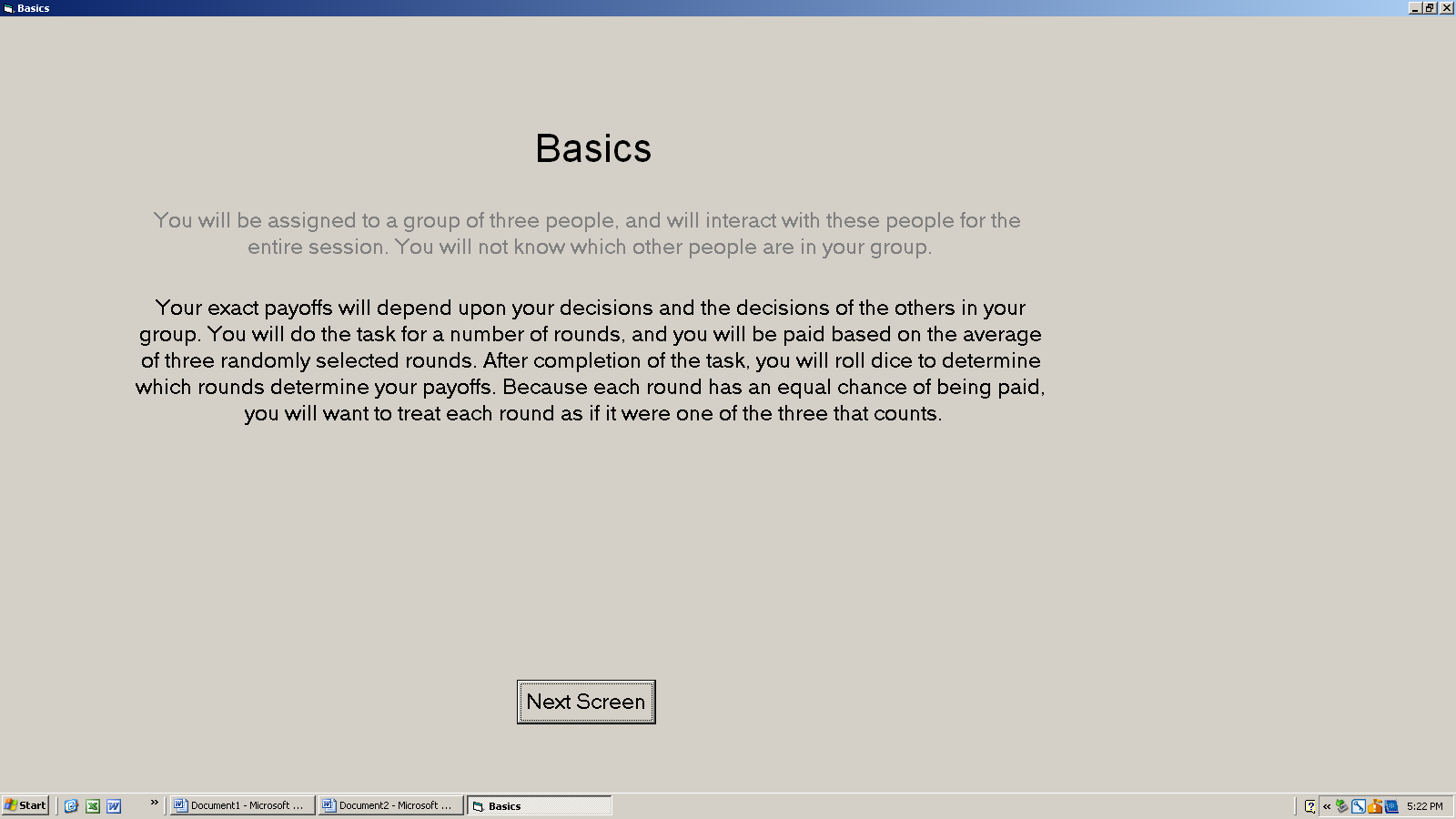


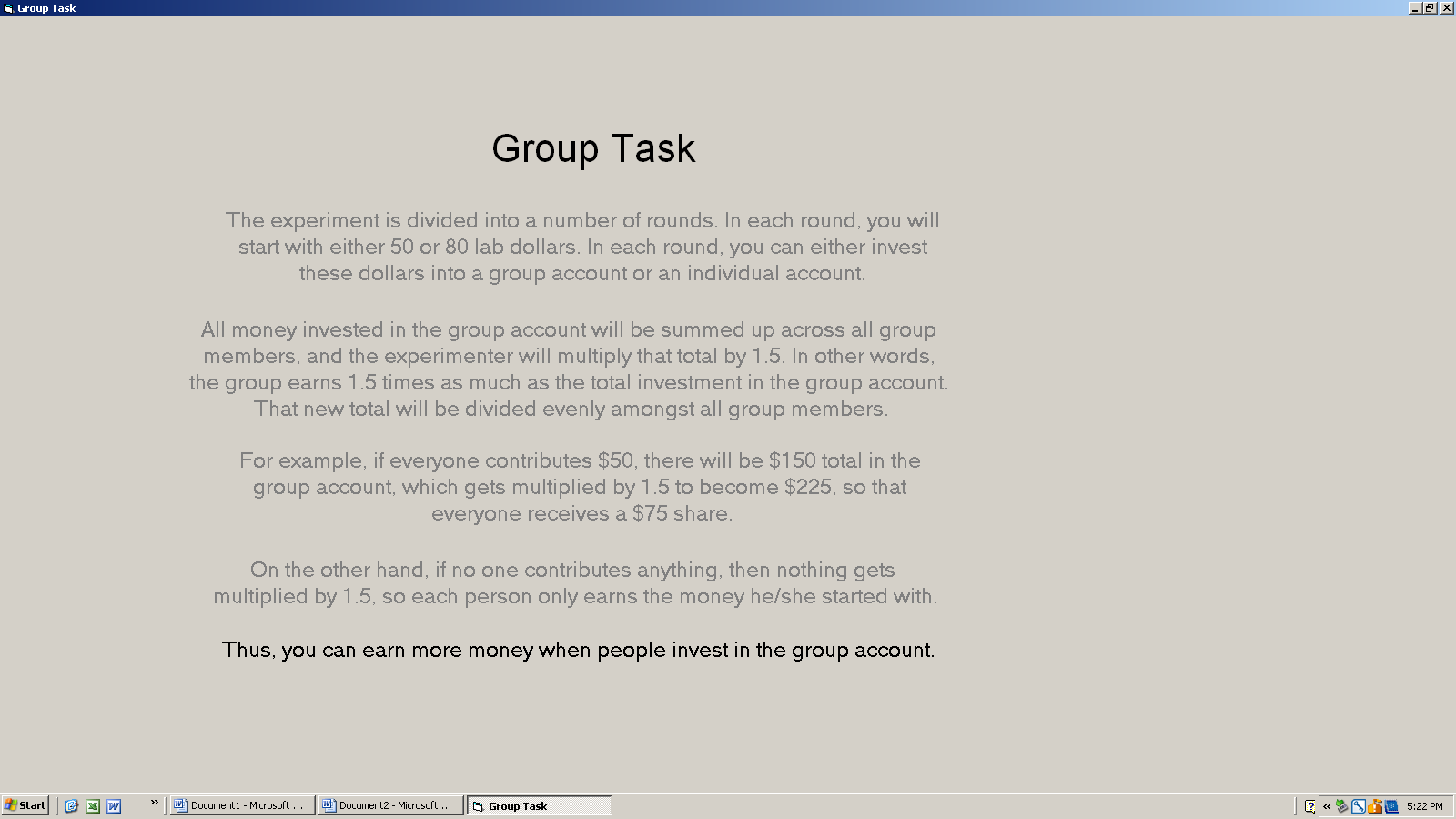


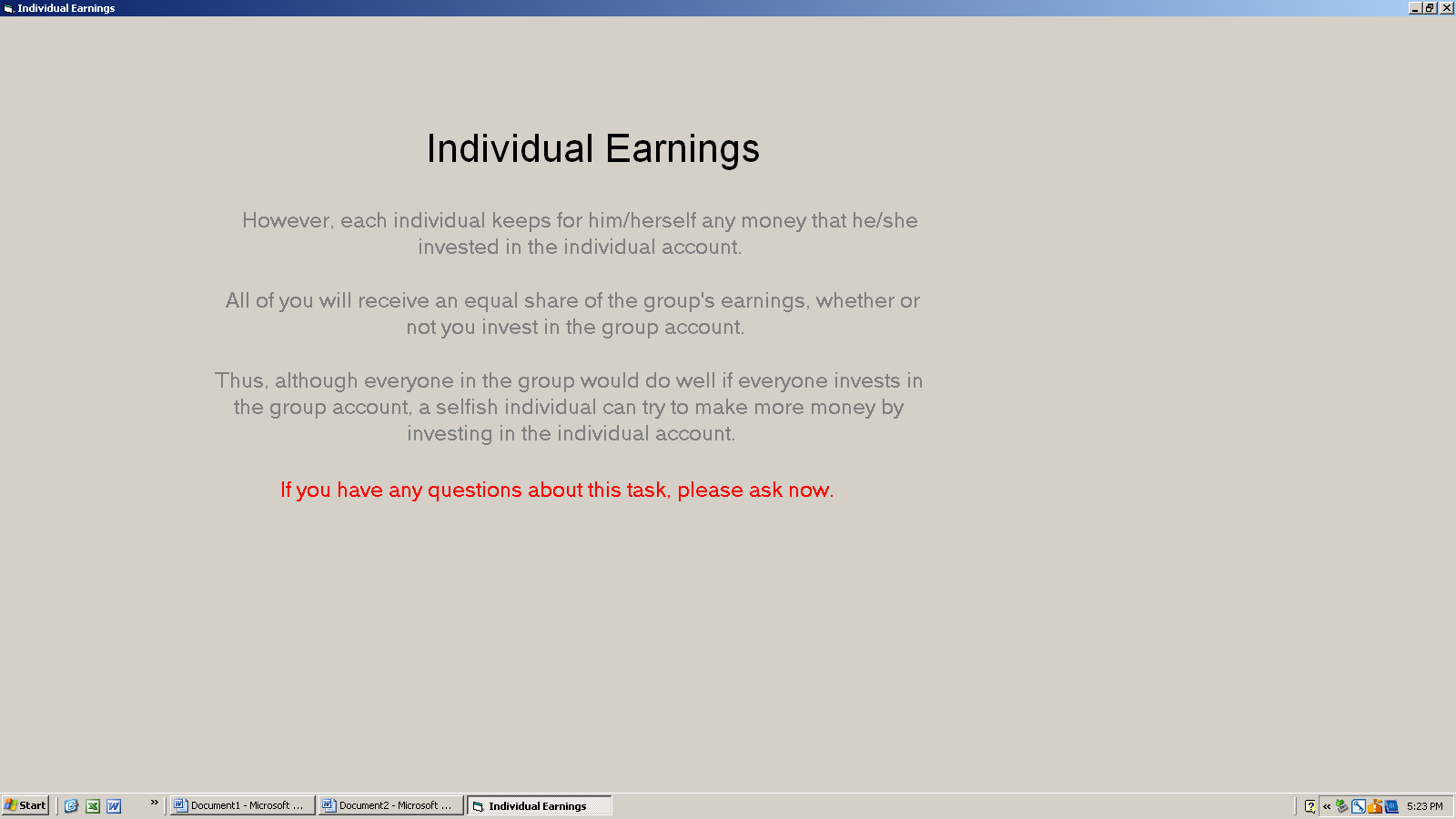


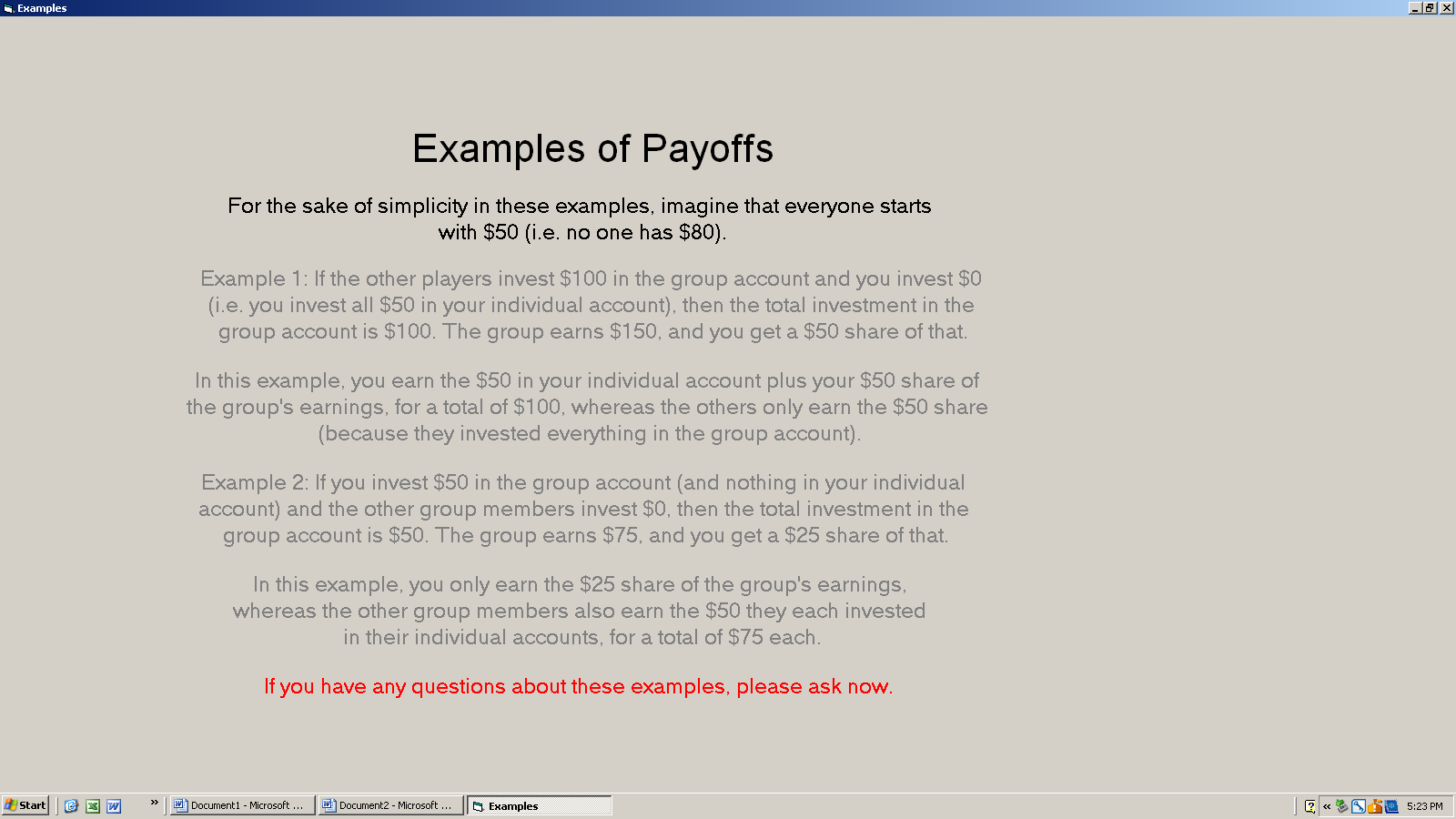


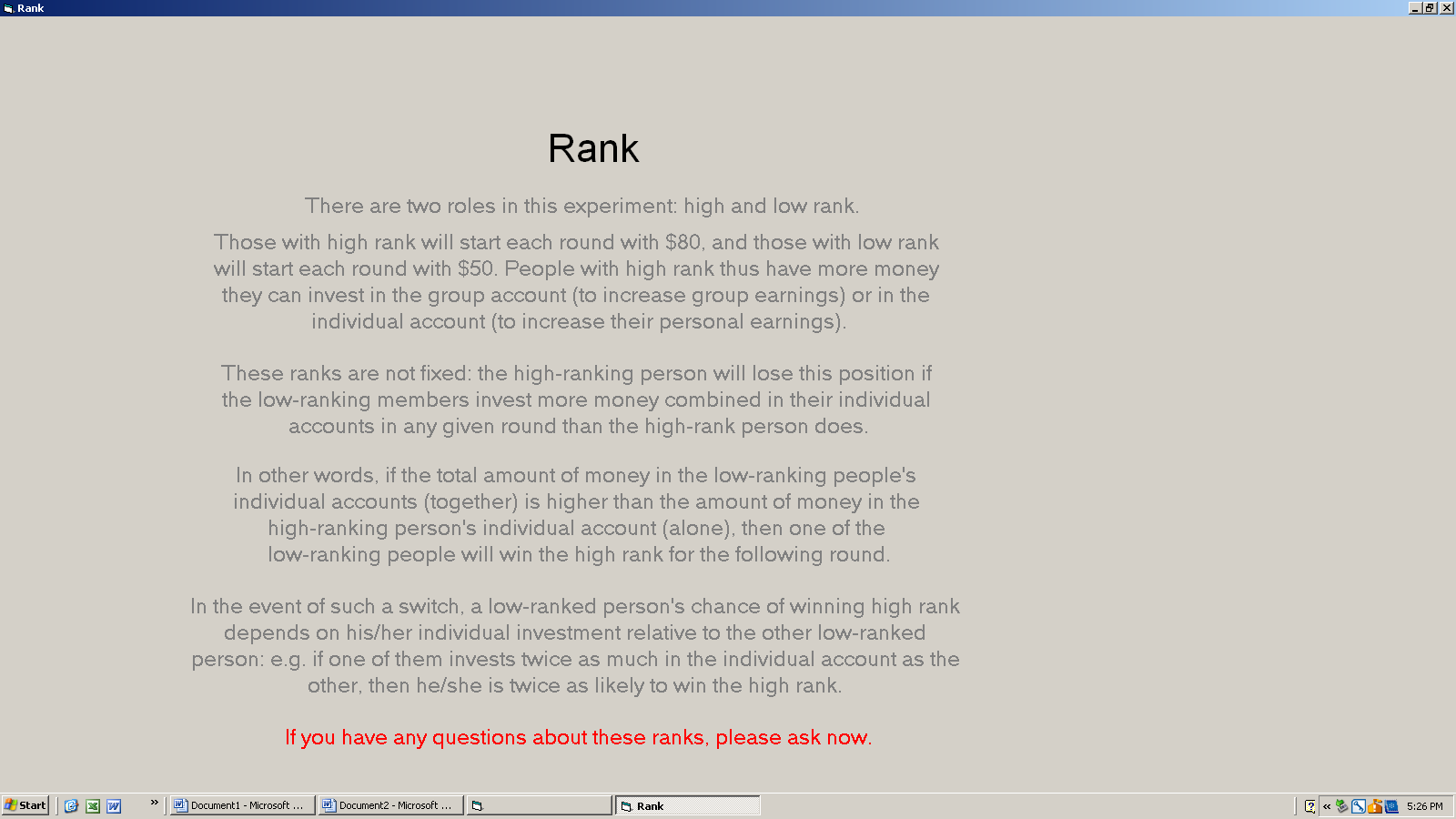


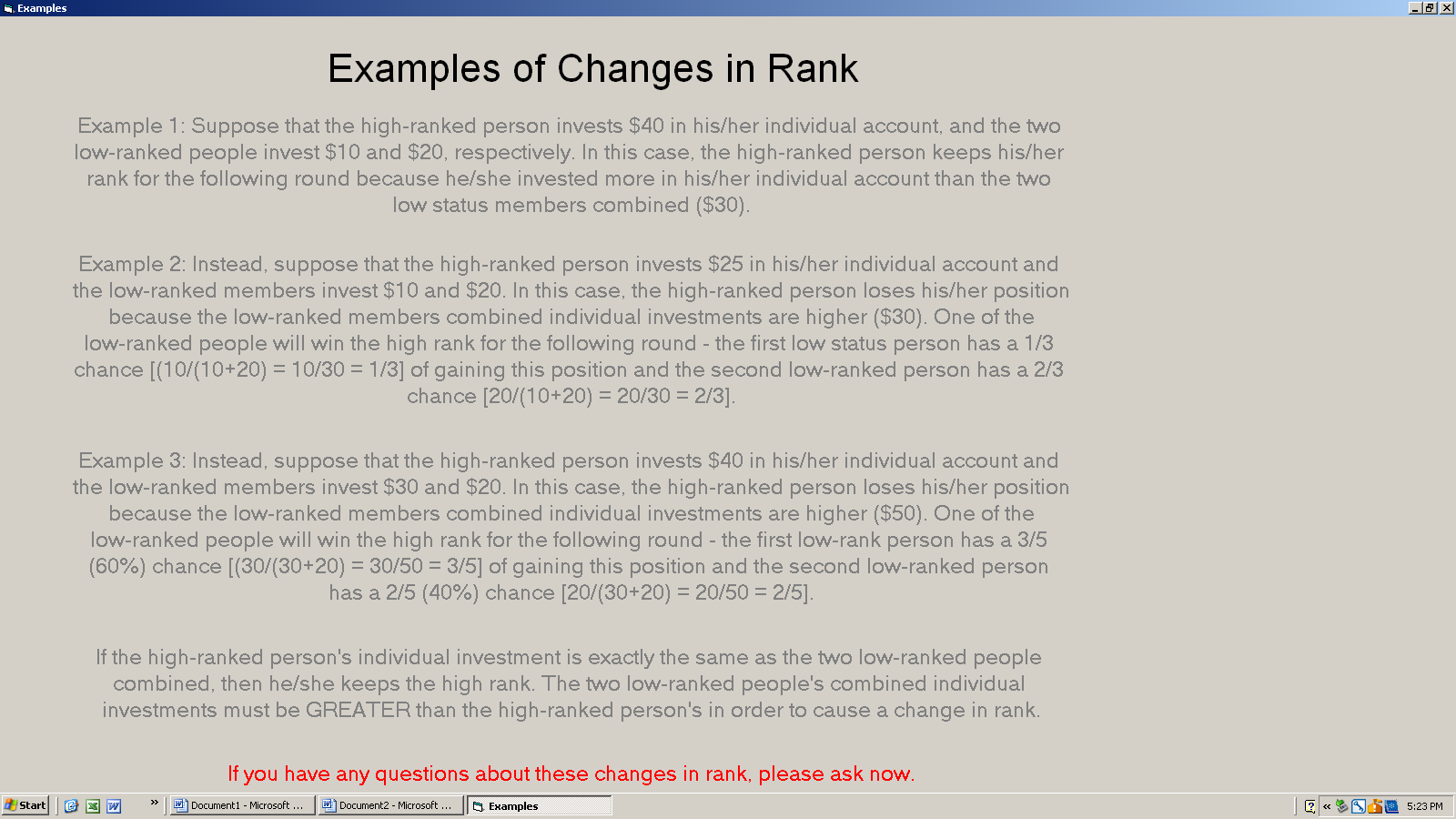


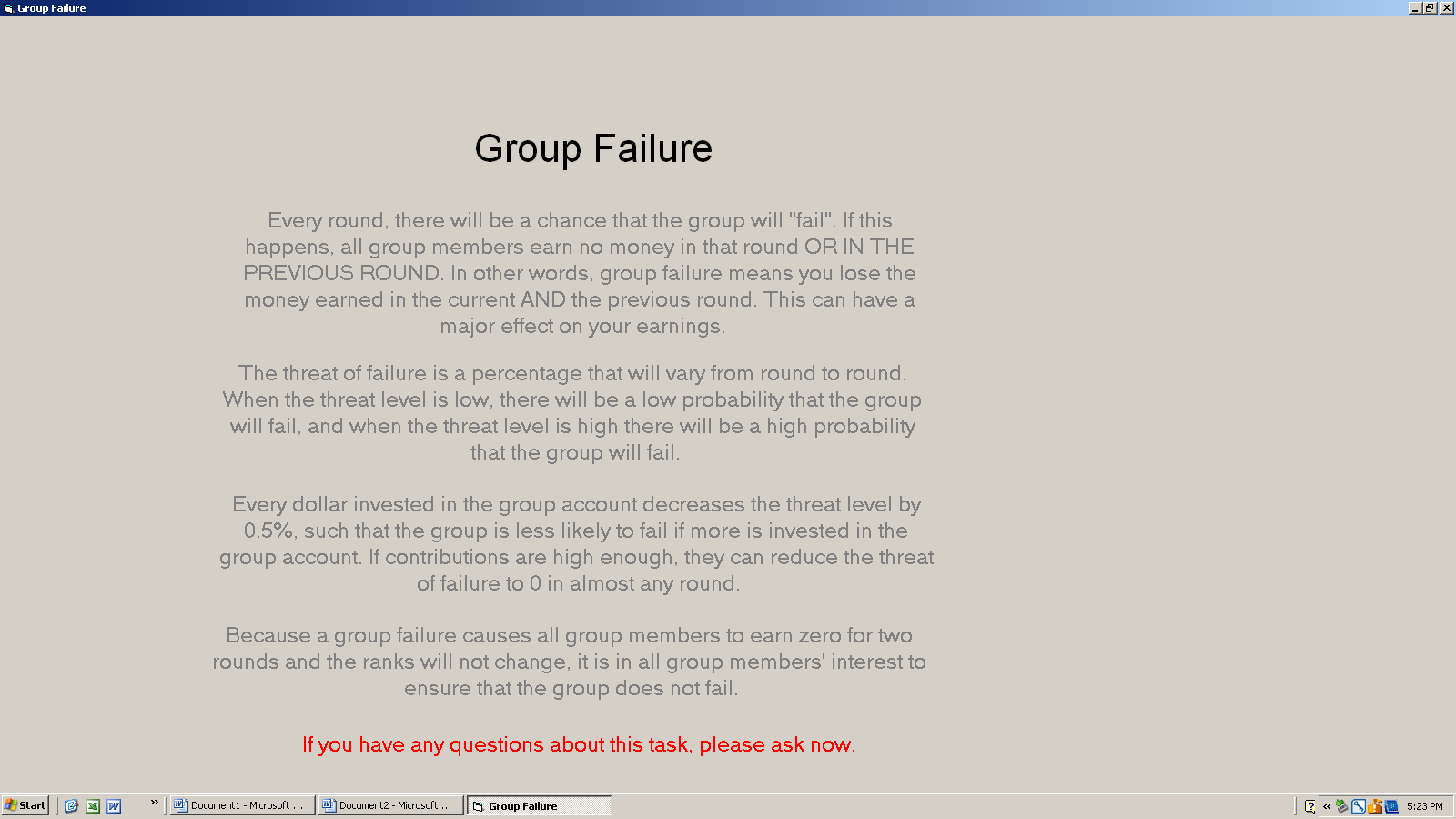


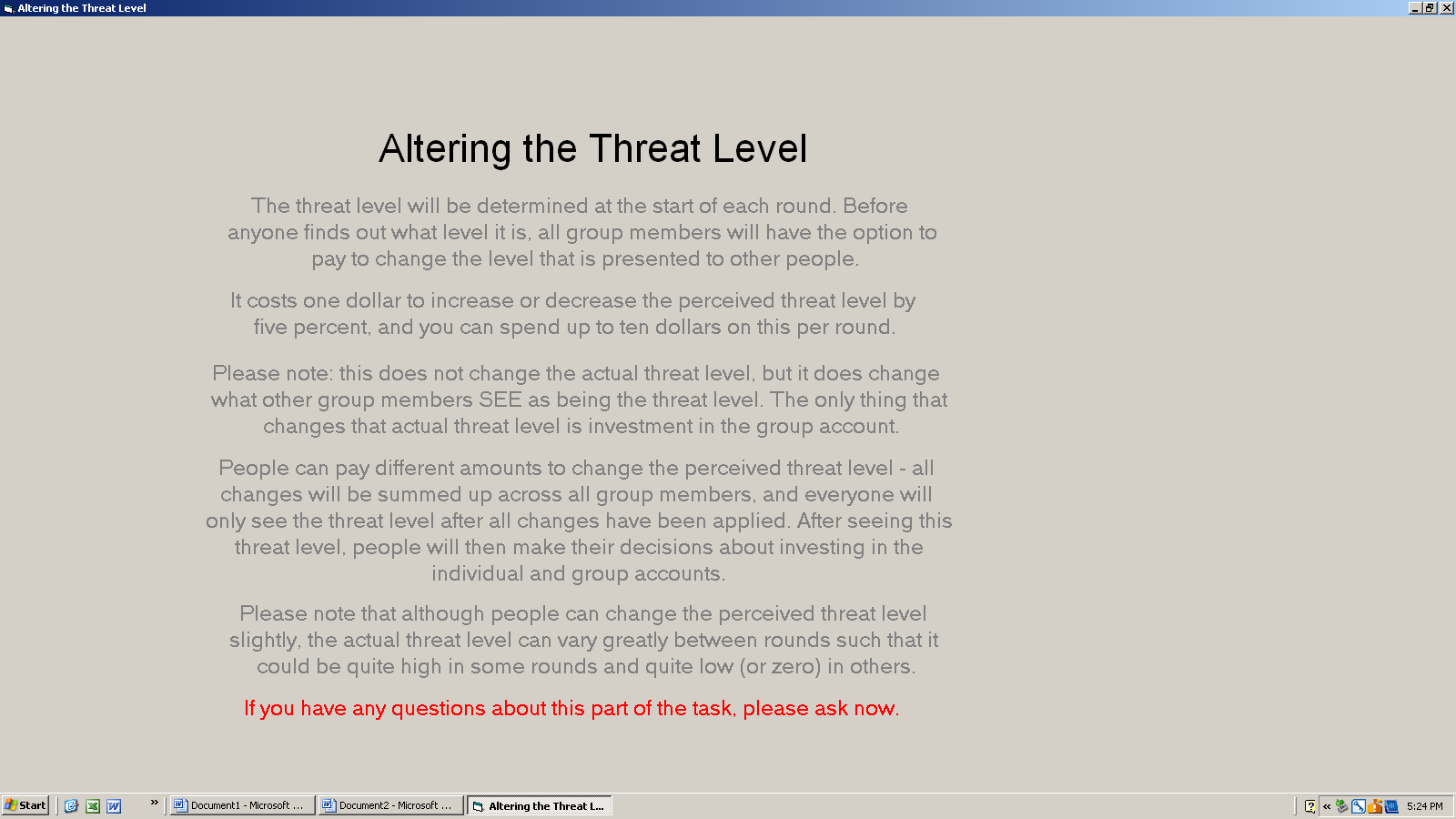


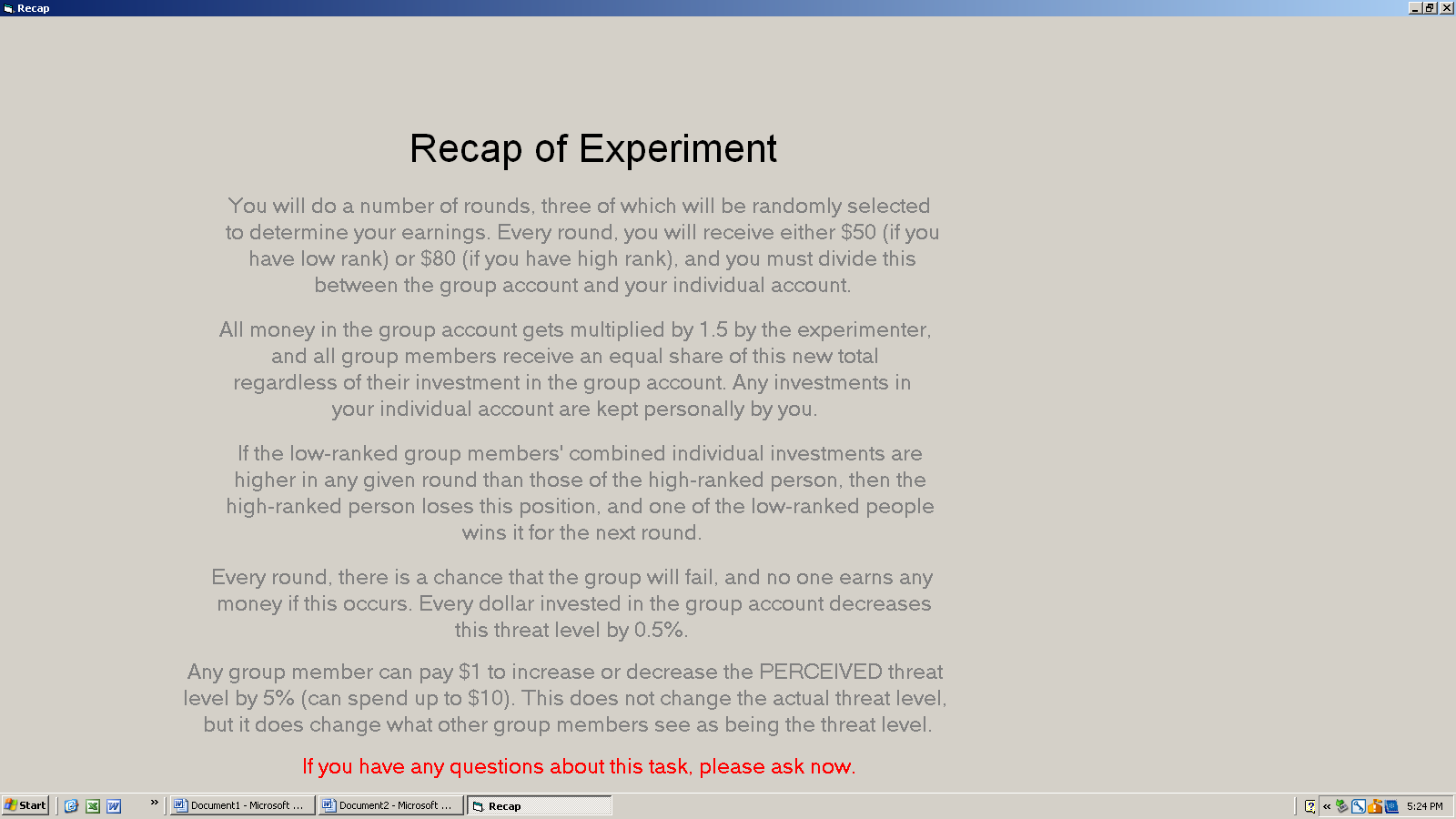


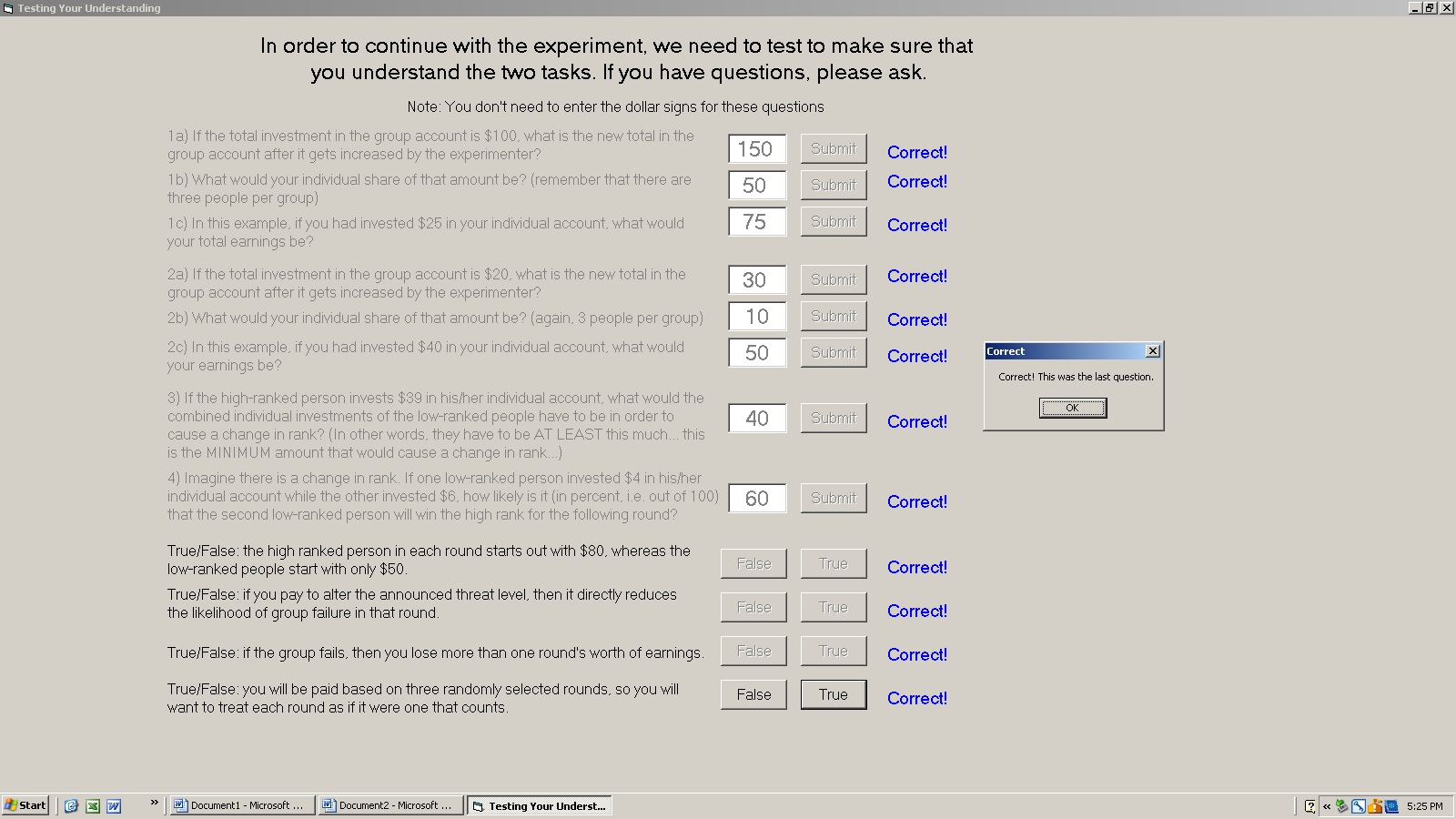


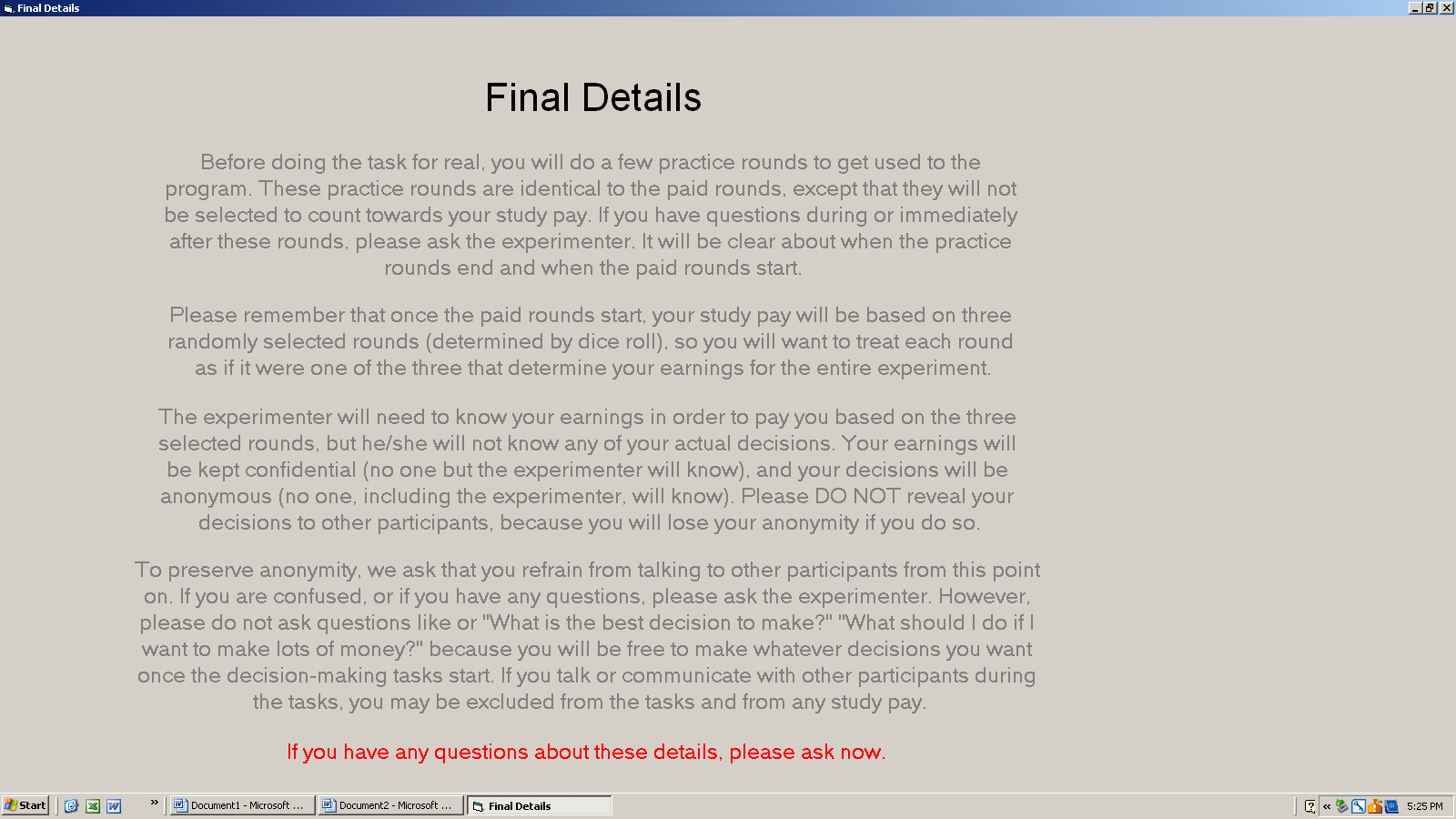


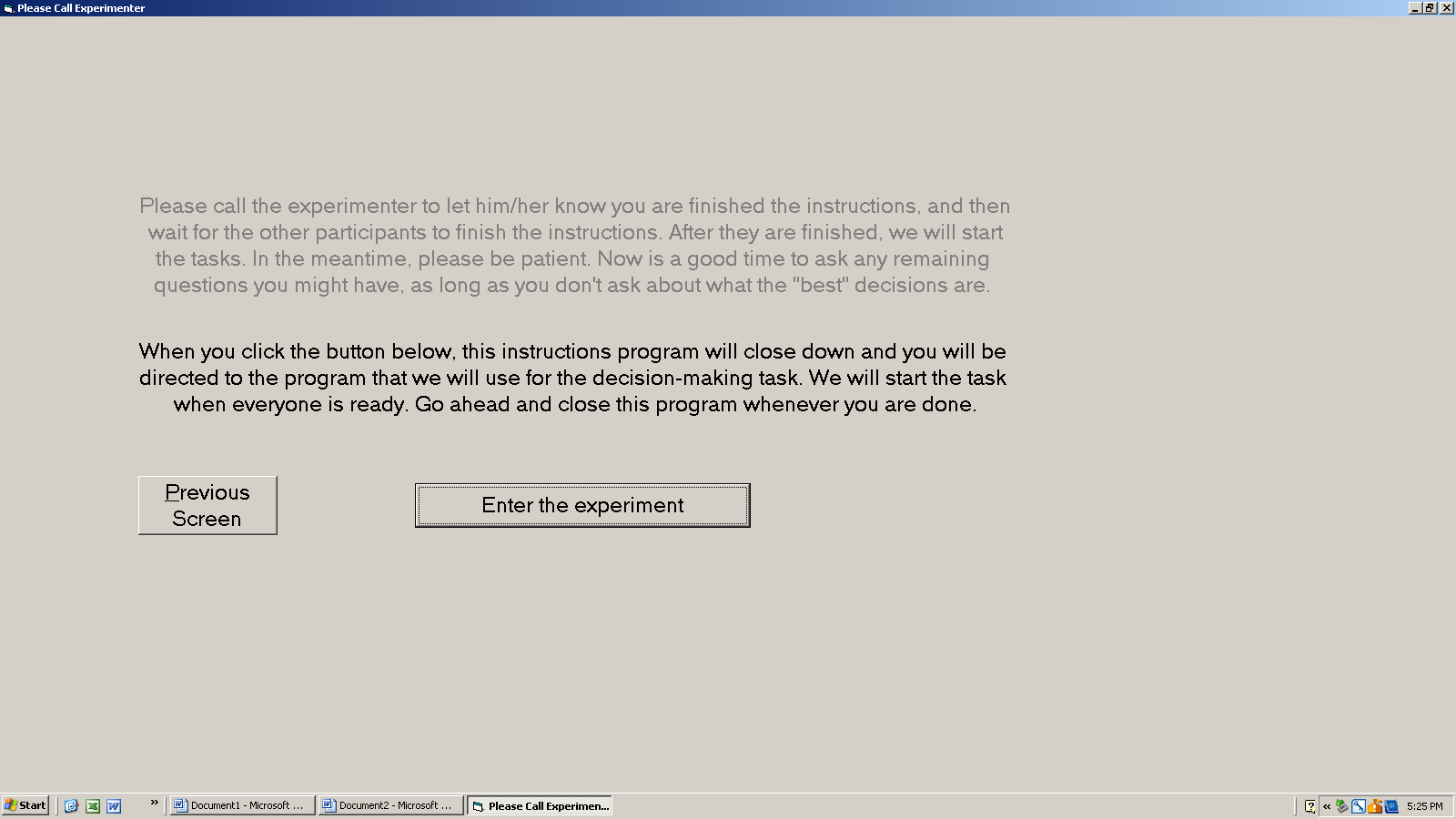

Supplement: Text S1 — Screenshots of the instructions for the experiment. (DOCX) [file pone.0073863.s001.docx]
