# Supplementary material for: Who Cries Wolf, and When? Manipulation of Perceived Threats to Preserve Rank in Cooperative Groups
Source: PLoS One. 2013 Sep 12;8(9):e73863. doi: 10.1371/journal.pone.0073863 (PMC3772075; doi:10.1371/journal.pone.0073863)
Supplement: Text S2 — Descriptive statistics of the average manipulation and contributions in Studies 1–3. (DOCX) [file pone.0073863.s002.docx]

Supporting Text S2: Descriptive Statistics, Studies 1-3

|  |  |  | Absolute Amount Contributed to Group Fund | | Percent of Endowment Contributed to Group Fund | | Absolute Amount Spent Increasing the Threat Level | | Percent of Endowment Spent Increasing the Threat Level | |
| --- | --- | --- | --- | --- | --- | --- | --- | --- | --- | --- |
|  | Rank | Additional Conditions | |  |  |  |  |  |  |  |
|  |  |  |  |  |  |  |  |  |  |  |
| Study 1 | High Rank | NA | 16.69 | (22.83) | 20.86 | (28.53) | 1.98 | (3.69) | 2.48 | (4.61) |
|  |  |  |  |  |  |  |  |  |  |  |
|  | Low Rank | NA | 16.28 | (17.80) | 32.55 | (35.59) | 0.46 | (2.46) | 0.92 | (4.92) |
|  |  |  |  |  |  |  |  |  |  |  |
|  |  |  |  |  |  |  |  |  |  |  |
| Study 2 | High Rank | Contestable Rank | 22.66 | (23.82) | 28.33 | (29.78) | 1.85 | (3.91) | 2.31 | (4.89) |
|  |  | Random Rank | 35.54 | (27.59) | 44.42 | (34.49) | 1.33 | (3.41) | 1.66 | (4.26) |
|  |  |  |  |  |  |  |  |  |  |  |
|  | Low Rank | Contestable Rank | 21.38 | (18.16) | 42.76 | (36.31) | 0.24 | (2.42) | 0.48 | (4.85) |
|  |  | Random Rank | 26.13 | (18.97) | 52.25 | (37.94) | 0.60 | (2.35) | 1.19 | (4.70) |
|  |  |  |  |  |  |  |  |  |  |  |
|  |  |  |  |  |  |  |  |  |  |  |
| Study 3 | High Rank | Extra Power | 29.22 | (25.35) | 36.52 | (31.68) | 0.53 | (3.22) | 0.66 | (4.02) |
|  |  | Baseline | 24.75 | (21.79) | 30.94 | (27.24) | 0.79 | (3.30) | 0.99 | (4.12) |
|  |  |  |  |  |  |  |  |  |  |  |
|  | Low Rank | Extra Power | 22.98 | (18.61) | 45.97 | (37.21) | 0.17 | (2.21) | 0.34 | (4.42) |
|  |  | Baseline | 22.56 | (18.31) | 45.12 | (36.62) | 0.35 | (3.02) | 0.70 | (6.03) |
|  |  |  |  |  |  |  |  |  |  |  |

Table S1: Means (and standard deviations) of amounts contributed to the group fund and spent on net increases in the threat level, in both absolute lab dollars and as a percent of endowment (high- and low-ranking endowments were L$80 and L$50, respectively).
