# Supplementary material for: Who Cries Wolf, and When? Manipulation of Perceived Threats to Preserve Rank in Cooperative Groups
Source: PLoS One. 2013 Sep 12;8(9):e73863. doi: 10.1371/journal.pone.0073863 (PMC3772075; doi:10.1371/journal.pone.0073863)
Supplement: Text S3 — Average contributions to the group fund across rounds. (a) Study 1. (b) Study 2. (c) Study 3. (DOCX) [file pone.0073863.s003.docx]

Supplementary Text S3: Contributions Over Time, Studies 1-3

We present the trends in cooperation over time for each study. Figure S1a-c includes the proportion of endowment contributed to the group fund each round, by high/low rank and experimental manipulations (the Contestable Rank Condition for study 2, Figure S1b, and the Extra Power Condition for study 3, Figure S1c). As described in the main text, contributions declined significantly across rounds in all three studies, and those with high rank contributed a significantly smaller percentage of their endowment than did those with low rank in all three studies.

Figure S1: Average proportion of endowment contributed to the group fund in: a) Study 1; b) Study 2; and c) Study 3.
