# Supplementary material for: Who Cries Wolf, and When? Manipulation of Perceived Threats to Preserve Rank in Cooperative Groups
Source: PLoS One. 2013 Sep 12;8(9):e73863. doi: 10.1371/journal.pone.0073863 (PMC3772075; doi:10.1371/journal.pone.0073863)
Supplement: Text S4 — Effects of group failure on manipulation and contributions in Studies 1–3. (DOCX) [file pone.0073863.s004.docx]

Supplementary Text S4: Effects of Group Extinction, Studies 1-3

In this section, we present some additional analyses regarding the effects of group failure on contribution and threat manipulation across three studies. We investigate whether participants tended to change their behavior in response to a group failure. These analyses do not directly test the hypotheses, but may be of interest to other researchers. To evaluate the effects of group failure, we estimate the models with variables indicating (1) whether the group failed on the preceding round (i.e. a lagged measure of group failure) and (2) the interaction of the lagged group failure measure and the rank measure. This allows us to test whether individuals change their contribution or manipulation behavior in response to a group failure on the preceding round, and whether the effect of failure differs for group members in the high and low-ranking positions. The results for percent of endowments contributed, and percent of endowment spent on manipulation, are shown in Table S3.

Table S3. Multilevel model of percent of endowment (and standard error) invested in contribution and manipulation, controlling for group failure on prior round and interaction of failure and rank, across all three studies. See study descriptions for variable descriptions and sample sizes. + *p* < 0.10; * *p* < .05; ** *p* < .01

|  |  | Study 1 | | Study 2 | | Study 3 | |
| --- | --- | --- | --- | --- | --- | --- | --- |
|  |  | Contribution | Manipulation | Contribution | Manipulation | Contribution | Manipulation |
| Fixed Effects | |  |  |  |  |  |  |
|  | Rank | -3.39+ | 0.92** | -7.07** | 1.05** | -2.36* | 0.35* |
|  |  | (1.87) | (0.31) | (1.03) | (0.16) | (1.13) | (0.16) |
|  | Perceived Threat | .31** |  | 0.34** |  | 0.20** |  |
|  |  | (0.02) |  | (0.01) |  | (0.02) |  |
|  | Round | -0.28* | 0.01 | -0.14** | 0.01* | -0.11** | 0.01 |
|  |  | (0.13) | (0.02) | (0.04) | (0.01) | (0.04) | (0.01) |
|  | Contestability |  |  | -12.39** | -0.22+ |  |  |
|  |  |  |  | (0.85) | (0.13) |  |  |
|  | Extra Power |  |  |  |  | 3.13** | -0.35** |
|  |  |  |  |  |  | (0.89) | (0.13) |
|  | Group Failed | 2.23 | -0.31 | -0.21 | -0.30 | 0.00 | -0.10 |
|  | in Prior Round | (2.31) | (0.38) | (1.36) | (0.21) | (1.41) | (0.20) |
|  | Rank x Group Failed | -2.79 | 0.58 | 0.38 | -0.24 | -3.21 | -0.02 |
|  | in Prior Round | -3.93 | (0.65) | (2.32) | (0.35) | (2.43) | (0.34) |
|  | Constant | 18.10** | 1.11** | 35.57** | 0.79** | 32.88** | 0.56+ |
|  |  | (0.037) | (0.41) | (3.59) | (0.30) | (2.42) | (0.30) |
| Random Effects | |  |  |  |  |  |  |
|  | Individual-level | 13.28** | 2.30** | 15.20** | 2.53** | 16.93* | 2.68* |
|  | random errors | (1.70) | (0.20) | (1.40) | (0.20) | (7.19) | (1.12) |
|  | Group-level | 12.41** | 0.00 | 16.70** | 0.00 | 6.74 | 0.28 |
|  | random errors | (2.8) | (0.60) | (2.70) | (0.60) | (6.24) | (0.86) |

`

As shown in Table S3, neither the main effect of lagged group failure, nor the interaction of this term with rank, is significant in any of the models. When we analyzed absolute amounts spent instead of proportion of endowment (not shown in the table), we did find a marginally significant, negative Rank x Group Failure interaction for the absolute amount spent increasing the threat level in study 2; this negative interaction was also significant in study 3. This suggests that high-ranking individuals manipulate somewhat *less* in absolute terms following a failure. If manipulation were a prosocial behavior to prevent group failure, then we should have expected *more* manipulation following group failure (not less), so this finding provides an additional argument against manipulation being a “benevolent lie” intended to help the group.
