# Supplementary material for: Who Cries Wolf, and When? Manipulation of Perceived Threats to Preserve Rank in Cooperative Groups
Source: PLoS One. 2013 Sep 12;8(9):e73863. doi: 10.1371/journal.pone.0073863 (PMC3772075; doi:10.1371/journal.pone.0073863)
Supplement: Text S5 — Effect of manipulation on earnings efficiency in Studies 1–3. (DOCX) [file pone.0073863.s005.docx]

Supplementary Text S5: Efficiency, Studies 1-3

In this section, we examine the correlation between investment in manipulation and earnings efficiency. These findings do not directly test a hypothesis, but explore whether manipulation is associated with an increase in group welfare or whether the cost of manipulation outweighs any collective gain. In these analyses, we created a measure of efficiency at the group level by dividing the total group earnings each round by the maximum possible group earnings each round (L$270). For each of the three experiments, we regressed this efficiency measure on the amount spent increasing the threat per round, any experimental manipulations (the Contestable Rank Condition for study 2, and the Extra Power Condition for study 3), and a control for round. The unit of analysis was the group-round (e.g. Study 1 included 22 groups over 20 rounds for a total of 440 observations). Standard errors are clustered by group ID. The results are shown in Table S2.

Table S2. OLS regression of group-level efficiency each round (total earnings/maximum earnings) on amount spent increasing the threat, round, the Contestable Rank Condition (study 2 only) and the Extra Power Condition (study 3 only), across all three studies. The unit of analysis is the group-round. Standard errors (in parentheses) are clustered at the group level. + *p* < 0.10; * *p* < .05; ** *p* < .01

|  |  | Study 1 | Study 2 | Study 3 |
| --- | --- | --- | --- | --- |
|  | Total Spent Increasing | 0.03 | -0.02* | 0.01 |
|  | Threat Level | (0.02 ) | (0.01) | (0.01) |
|  | Round | -0.01 | 0.00 | 0.00 |
|  |  | (0.00) | (0.00) | (0.00) |
|  | Contestability |  | (-0.15)** |  |
|  |  |  | (0.03) |  |
|  | Extra Power Condition |  |  | 0.10** |
|  |  |  |  | (0.03) |
|  | Constant | 0.51** | 0.63** | 0.47** |
|  |  | (.06) | (0.05) | (0.03) |
|  | Number of Observations | 440 | 1,280 | 1,440 |
|  |  |  |  |  |

In studies 1 and 3, the amount spent on manipulation was positively but non-significantly related to group efficiency (p > .14 & p > .25, respectively). In Study 2, the effect was negative and significant (p < 0.05), such that groups with more manipulation had lower efficiency. To examine this negative effect in Study 2 more closely, we ran the analyses separately by condition. In the Contestable Rank condition (which is a near-replication of Study 1), we found a non-significant, positive effect of investment in manipulation (p > 0.64), similar to our findings for Study 1. In the Random Rank condition, we found a significant, negative effect of threat manipulation, such that groups were less efficient in rounds with greater manipulation (p < 0.001).

The experimental conditions in Studies 2 and 3 have an effect on group efficiency. The Contestable Rank Condition (Study 2) has a significant, negative effect on efficiency, such that competition over rank leads to less efficient outcomes than randomly assigning rank. In Study 3, we find that the Extra Power Condition significantly increases efficiency. As noted in the main text, however, this gain in efficiency is tempered by the fact that the high-ranking group member claims 57% of the group’s output on average, while contributing 37% of the input to the public good; in other words, the high-ranking member is claiming most or all of this gain in efficiency.

Based on these analyses, we cannot conclude that threat manipulation increases group efficiency, and may decrease it (Study 2). Although threats increase contribution, directly contributing to the group appears to more effectively increase contribution. We do not claim that threat manipulation is never socially efficient, but we do not find evidence that it is efficient in our research.
