# Supplementary material for: Who Cries Wolf, and When? Manipulation of Perceived Threats to Preserve Rank in Cooperative Groups
Source: PLoS One. 2013 Sep 12;8(9):e73863. doi: 10.1371/journal.pone.0073863 (PMC3772075; doi:10.1371/journal.pone.0073863)
Supplement: Text S6 — Detailed analysis of the division of the public good by the high-ranking player in Study 3. (DOCX) [file pone.0073863.s006.docx]

Supplementary Text S6: Division of the public good in Study 3

In this section, we investigate factors affecting how the participant in the high-ranking role distributed the public good. Individuals who allocate resources on behalf of a group face a tension between two alternatives. On one hand, many people subscribe to the concept of equity, or the notion that one should be rewarded in proportion to their contributions to the group. On the other hand, many people also seek to maximize their own rewards, which encourages low contributions but high self-allocations (e.g., Schroeder et al 2003).

We focus on two variables: the proportion of the public good that the high-ranking individual allocated to themselves, and the proportion of the contributions to the public good the high-ranking individual made. We call this latter measure the “high-ranking equitable share” (HRES), because it is also the proportion of the public good that the high-ranking individual would receive in a purely equitable system in which allocations are proportional to contributions.

To provide an overview of the pattern of contribution and allocations, Figure S2 shows the average proportion of the public good that the high-ranking participant allocated to each individual, by role. The figure also shows each individual’s proportional contribution to the public good, which corresponds to their “equitable share”. As can be seen in Figure S2, the high-ranking individual tended to allocate 57% of the public good to themselves, while contributing about 37% of the input to the public good. The difference between the high-ranking individual’s actual and equitable share is significant greater than zero (*b* = .20, *z* = 7.70, *p* < 0.001, tested using an intercept-only model with this difference as the dependent measure). Thus, individuals in high-ranking roles reward themselves out of proportion to their inputs. In contrast, high-ranking individuals tend to under-reward those of low rank. Low-ranking participants one and two (numbered arbitrarily) tended to contribute 30-33% of the input to the public good, and receive about 20-22% of the resulting public good from the high-ranking individual (the differences between actual and equitable shares for both low-ranking participants are significant at *p* < 0.001).

To further explore the relationship between the amount the high-ranking participant allocates to self, and their equitable share, we estimated a multilevel model with the proportion the high-ranking participant allocates to self as the dependent measure, and the high-ranking participant’s equitable share as an independent variable. We also include five controls: the average amount the high-ranking participant contributed during the study, the total amount contributed by the group that round, the high-ranking participant’s investment in manipulation as a proportion of their endowment that round, the perceived threat that round, and round. The results are shown in Table S4.

Figure S2: Mean proportion (± s.e.) of the public good in Study 3 that was provided (light bars) and received (dark bars) by the high ranking participant and the two low ranking participants (it was arbitrarily determined which low ranking participant was assigned to be 1 or 2). High-ranking participants received a larger share than they provided, whereas low ranking participants received smaller shares than was equitable.

In Table S4, the intercept (b = 0.49, z = 8.26, p < 0.0001) indicates that, controlling for round and average contributions, high-ranking individuals tend to allocate about 49% of the public good to themselves, when their own equitable share is zero. The positive and significant equitable share measure indicates that, net of controls, high-ranking participants tend to allocate themselves an additional half percentage point of the public good for every additional percentage of the input they contribute (b = 0.50, z = 15.11, p < 0.0001).^[[1]](#footnote-1)^ That fact that the amount allocated to self increases at a lower rate than the equitable share means that, at high levels of relative contribution, the high-ranking participants actually allocates less to self than what they would receive under an equitable share. However, this only occurs when the high-ranking person is providing close to all of the input to the public good. The percent kept by the high-ranking members was also negatively correlated with their average contributions, (*b* = -.25, z = -2.08, p < 0.037), and positively correlated with round (b = .003, z = 2.70, p < 0.007), but not correlated with their own manipulation, the total group contributions, or the perceived threat level (all *p*s > .17).

Table S4. Multilevel model of the proportion of the public good high-ranking individuals allocated to self. Numbers represent the effect of a one unit change in the independent variables on the proportion the public good the high ranking individual kept each round. The model is based on 594 person-rounds, including only high-ranking individuals in the Extra Power condition when the group did not go extinct (because only these individuals had an opportunity to allocate the public good). + *p* < 0.10; * *p* < .05; ** *p* < .01

|  |  | Proportion |
| --- | --- | --- |
| Fixed Effects |  | Allocated to Self |
|  | High-Ranking | 0.50** |
|  | Equitable Share | (0.03) |
|  | Total Group | 0.00 |
|  | Contributions | (0.00) |
|  | Proportion of Endowment | -0.16 |
|  | Invested in Manipulation | (0.29) |
|  | Average Proportion of | -2.46* |
|  | Endowment Contributed | -0.12 |
|  | Perceived | 0.00 |
|  | Threat | 0.00 |
|  | Round | 0.003** |
|  |  | (0.00) |
|  | Constant | 0.49** |
|  |  | (0.06) |
| Random Effects |  |  |
|  | Individual-level | 0.18** |
|  | random errors | -0.02 |
|  | Group-level | 0.08* |
|  | random errors | -0.03 |

Figure S3 shows the proportion of the public good the high-ranking participant allocated to self, as a function of the proportion they invested in the public good. For comparison, we also include a line indicating the high-ranking participant’s equitable share. In general, high-ranking participants tend to allocate to themselves more than their equitable share. However, when the high-ranking participant provides 81-90% of the public good, they tend to reward themselves at a level roughly equal to their input. When high-ranking participants provide more than 90% of the public good, they tend to allocate approximately 77% of the public good to themselves.

Figure S3. Proportion of the public good in Study 3 claimed by high-ranking participants (solid line) as a function of the proportion of the public good they themselves provided. The dotted line represents an equitable share based on their contributions.

We also examine how participants’ earnings differ by rank and power condition, shown in Table S5. As a result of the unequal division of the public good, high-ranking members earn more in the Power Condition than the Baseline Condition (mean earnings L$81.03 vs. L$53.52. whereas low-ranking members earned similar amounts across conditions (L$37.40 vs. L$37.80). To test the significance of these trends, we estimated a multilevel model with earnings as the dependent measure, and Rank, Power, their interaction, and a control for round. The effect of rank indicates that high-ranking participants earn significantly more than low-ranking participants in the baseline condition (b = 15.37, z = 8.68, p < 0.0005). The effect of the power manipulation indicates that low-ranking participants’ earnings do not vary significantly by condition (p > .73). The Rank x Extra Power interaction is significant and positive, indicating that being in the high-ranking position has a greater effect on earnings in the power condition (b = 28.19, z = 11.41 p < 0.0005). This suggests that granting additional powers to privileged members tends to increase inequity in earnings.

Table S5. Multilevel model of earnings per round predicted by rank, power condition, round, and the rank x power condition interaction. Numbers represent the effect of a one unit change in the independent variables on the number of lab dollars earned that round. The model is based on 4,320 observations (108 participants across 40 rounds). + *p* < 0.10; * *p* < .05; ** *p* < .01

|  |  |  |
| --- | --- | --- |
| Fixed Effects |  | Earnings |
|  | Rank | 15.37** |
|  |  | (1.77) |
|  | Extra Power | -0.49 |
|  | Condition | (1.41) |
|  | Round | -0.00 |
|  |  | (0.05) |
|  | Rank x Extra Power | 28.19** |
|  | Condition | (2.47) |
|  | Constant | 37.83** |
|  |  | (2.44) |
| Random Effects |  |  |
|  | Individual-level | 2.61+ |
|  | random errors | (1.39) |
|  | Group-level | 11.72** |
|  | random errors | (1.53) |

Additional References:

Schroeder, D. A., Steel, J. E., Woodell, A., & Bembenek, A.(2003). Justice within social dilemmas. *Personality and Social Psychology Review*, *7*, 374-387.

1. The intercept and effect of HRES are very similar with the control variables omitted. [↑](#footnote-ref-1)
