# Supplementary material for: Who Cries Wolf, and When? Manipulation of Perceived Threats to Preserve Rank in Cooperative Groups
Source: PLoS One. 2013 Sep 12;8(9):e73863. doi: 10.1371/journal.pone.0073863 (PMC3772075; doi:10.1371/journal.pone.0073863)
Supplement: Text S7 — Analysis of order effects in Studies 2–3. (DOCX) [file pone.0073863.s007.docx]

Supplementary Text S7: Order Effects

The order of conditions had little effect on the results of studies 2 and 3 (reported briefly here, tables available upon request). We tested for order effects in Study 2 by adding variables for order (1 = Random Rank condition first), and the interaction effect of order, the Random Rank condition, and order and whether the participant was high or low rank. There were no main effects or interactions regarding the order of conditions in Study 2, either on manipulation or on contributions to the group fund.

We tested for order effects in Study 3 by adding variables for order (1 = Extra Power condition first), and the interaction effect of order and the Extra Power condition. The order of conditions had no effect on manipulation, nor did it interact with the power manipulation (all *zs* < 1.5, all *ps* > .15). However, the order of conditions did affect contributions to the public good: participants contributed more when the Extra Power condition came first (order effect: *b* = 12.7, *p* < 0.003). This effect was qualified by an Order x Condition interaction (*b* = -10.53, *p* < 0.003) such that when the Extra Power condition came first, participants primarily contribute more in the Baseline condition. Our interpretation of this order effect is that the higher contributions in the Extra Power condition carry over into the Baseline condition when the former comes first. Our other results are all unchanged if we include order in the analyses, with the exception that the main effect of Condition becomes stronger and significant (*b* = 6.8, *z* = 3.25, *p* = .001).
